# Synthesis, Spectroscopic Characterization, Structural Analysis, and Evaluation of Anti-Tumor, Antimicrobial, and Antibiofilm Activities of Halogenoaminopyrazoles Derivatives

**DOI:** 10.3390/antibiotics13121119

**Published:** 2024-11-22

**Authors:** Christina Zalaru, Florea Dumitrascu, Constantin Draghici, Marilena Ferbinteanu, Isabela Tarcomnicu, Maria Marinescu, Zenovia Moldovan, George Mihai Nitulescu, Rodica Tatia, Marcela Popa

**Affiliations:** 1 Department of Inorganic Chemistry, Organic, Biochemistry and Catalysis, Faculty of Chemistry, University of Bucharest, 90-92 Panduri Road, 030018 Bucharest, Romania; marilena.cimpoesu@g.unibuc.ro (M.F.); z_moldovan@yahoo.com (Z.M.); 2 “C.D. Nenitescu” Institute of Organic and Supramolecular Chemistry, Romanian Academy, 202 B Splaiul Independentei, 060023 Bucharest, Romania; fdumitra@yahoo.com (F.D.); cst_drag@yahoo.com (C.D.); 3 Cytogenomic Medical Laboratory, 35 Calea Floreasca, 014462 Bucharest, Romania; 4 Faculty of Pharmacy, “Carol Davila” University of Medicine and Pharmacy, Traian Vuia 6, 020956 Bucharest, Romania; george.nitulescu@umfcd.ro; 5 Department of Cellular and Molecular Biology, National Institute of Research and Development for Biological Sciences, 296 Splaiul Independenţei, 060031 Bucharest, Romania; 6 Department of Microbiology, Faculty of Biology, University of Bucharest, 1-3 Aleea Portocalelor St., 060101 Bucharest, Romania; marcela.popa@bio.unibuc.ro

**Keywords:** pyrazoles, heterocyclic molecules, cytotoxicity, anti-tumor, antimicrobial, biofilm formation

## Abstract

New haloaminopyrazole derivatives differing in the number of pyrazole nuclei **4a**–**f** and **5a**–**e**, respectively, were synthesized and characterized by ^1^H-NMR, ^13^C-NMR, IR, UV-Vis, and elemental analysis. The single-crystal X-ray diffraction method was used to describe compounds **4a** and **5d**. When tested on normal NCTC fibroblasts in vitro, the newly synthesized derivatives were shown to be non-cytotoxic at a dosage of 25 μg/mL. Two compounds **4a** and **5d** showed a high degree of biocompatibility. From the two series of compounds tested on HEp-2 human cervical carcinoma cells, compound **5d** showed a more pronounced antiproliferative effect. Gram-positive strains of *Staphylococcus aureus* ATCC25923, *Enterococcus faecalis* ATCC29212, Gram-negative strains of *Pseudomonas aeruginosa* ATCC27853, and strains of *Escherichia coli* ATCC25922 were used to test the newly synthesized compounds antibacterial and antibiofilm properties. Among the studied pyrazole compounds, 2 compounds **4a** and **5a** with fluorine content on the phenyl ring and 4 compounds **4b**, **4e**, **4f**, and **5b** with chlorine content on the phenyl ring were noted, which proved to be the most active compared with the two reference drugs, metronidazole and nitrofurantoin. The six compounds showed a broad spectrum of action against all four tested bacterial strains, the most active being compound **4b**, with a chlorine atom in the “4” position of the phenyl nucleus and a MIC of 460 μg/mL. Compounds **4a** and **5a** showed the best antibiofilm activity against the bacterial strain *Staphylococcus aureus* ATCC25923, with an MBIC of 230 μg/mL.

## 1. Introduction

The pyrazole nucleus is a key compound in pharmaceutical and organic synthesis, having a variety of therapeutic properties, such as anti-tumor [1,2,3,4,5,6,7,8,9,10,11,12], anti-inflammatory [6,12,13,14,15,16], antibacterial [17,18,19,20], anticonvulsant [21], antimalarial [22], and local anesthetic [23,24].

Another applicability of pyrazole is coordination chemistry. The pyrazole nucleus is used as a ligand in organometallic chemistry, being obtained by synthesis from formylmenthone and then coordinated [25,26]. From a structural point of view, the pyrazole nucleus has peculiarities of coordinating, namely: the protonated N1 atom will coordinate through N2 as a monodentate ligand. On the other hand, N1 can be easily deprotonated to form the pyrazolate anion, which can also coordinate with metal ions as a monodentate ligand [27]. The importance of essential metal ions is known, which contribute to biochemical processes with a vital role, while non-essential metal ions are used as diagnostic agents or drugs in therapies for various diseases [28]. Coordination chemistry, through research efforts, demonstrates the excellent bioactivity of transition metal ions; these are being used as anticancer agents compared to free, uncoordinated ligands [28]. The complex combinations of the pyrazole nucleus with metal ions bring a new and effective approach to cancer chemotherapy [28]. Especially the heterocyclic compounds, which contain nitrogen atoms, through their pharmacophoric structures, are selective and powerful anticancer agents, in many cases being approved anticancer drugs.

Metal complexes containing N-donor ligands, such as pyrazole derivatives, have demonstrated the most effective biological activities, which are attributed to the electronic interactions between the π electrons of the heterocyclic rings and the metal ions [28]. Also noteworthy is the antibacterial activity of heterocyclic metal complexes, demonstrated against several bacterial strains both in vitro and in vivo, as promising antibacterial agents [29].

An imbalance that can be found in cell proliferation is a cause of the appearance of some forms of cancer. Drugs that act to kill aberrant cells are considered promising antiproliferative agents in eradicating this scourge [30].

Due to the dramatic increase in multi-resistant microbial infections, new antimicrobial agents can intervene to stop these infections. Pyrazoles are an important component in obtaining antibacterial drugs [31,32].

Consequently, the development of new innovative drugs in the treatment of cancer and antimicrobial infections is topical [33,34,35].

The presence of chlorine or fluorine atoms in the 4-position of the phenyl ring, which is attached to the 4-aminopyrazole position, exhibits maximum antiproliferative activity against MCF-7 and B16-F10 cancer cell lines (murine carcinoma) [36].

Compounds containing halogen atoms grafted onto the aromatic rings can be considered anti-tuberculosis agents because the halogen atoms, through their presence, contribute to passive diffusion through the very impenetrable mycobacterial cell wall, being able to modulate lipophilicity [37].

**Figure 1:**
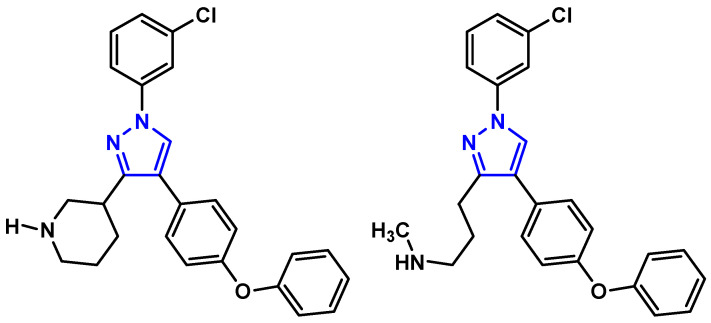
Pyrazole derivatives (R_2_ is 2-, 3-4-piperidyl).

Derivatives of aminopyrazoles that also contain halogen atoms grafted on aromatic rings demonstrate that they have antibacterial activity (Figure 1) [38].

We previously synthesized alkyl derivatives of pyrazole [34], respectively pyrazolo-benzimidazoles [35], which showed strong antimicrobial activity because in the first case, the length of the alkyl chain attached to the pharmacophore pyrazole ring prevailed, and in the second case, due to the presence of rings functionalized hybrid heterocyclics [34,35].

**Figure 2:**
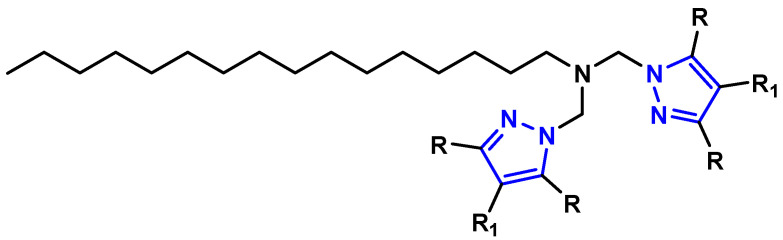
Alkylaminopyrazoles derivatives. Where R = H, CH_3_; R_1_ = H, I, NO_2_.

**Figure 3:**
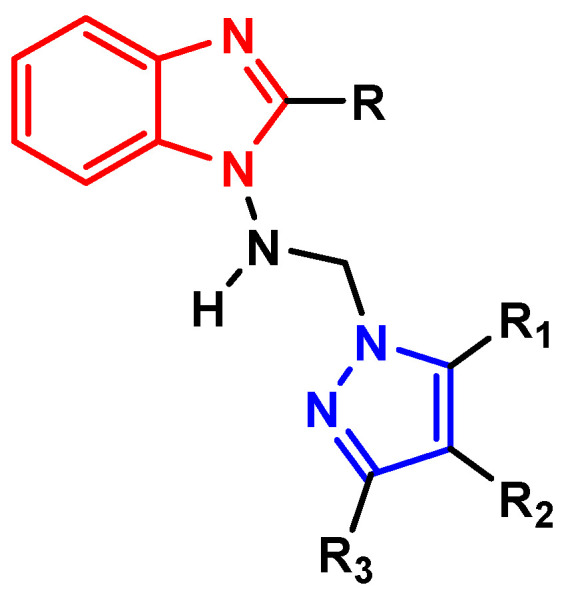
Pyrazolo-benzimidazole derivatives. Where R = H, CH_3_; R_1_ = R_3_ = H, CH_3_; R_2_ = H, I, NO_2_.

Figure 2 and Figure 3 show the structure of the previously reported compounds and the minimum inhibitory concentration.

The compounds showed the most significant activity against *Bacillus subtilis* with MIC values between 0.007 and 0.062 µg/mL versus erythromycin as standard (Figure 2) [34].

The compounds showed antibacterial activity against *Staphylococcus aureus* with MIC values between 190 and 1560 µg/mL versus metronidazole as standard (Figure 3) [35].

The presence of amino groups and halogen atoms located on the phenyl and pyrazole rings proved to be antitumor and antibacterial agents [36,38].

Starting from these structural features, the aim of this study consisted of the design of new structures that would present the pharmacophore pyrazole ring with the nitro group in position “4”, but also the phenyl ring functionalized with halogen atoms and the amino group. The structure as a whole should present both activity antitumor as well as antimicrobial.

The objectives of this study consist of the synthesis of new pyrazole derivatives starting from these structural considerations, the physico-chemical characterization, and testing for their biological activity. At the same time, we proposed to analyze and characterize the potential crystallographic structures of these pyrazole compounds.

## 2. Results

### 2.1. Chemistry

**Scheme 1:**
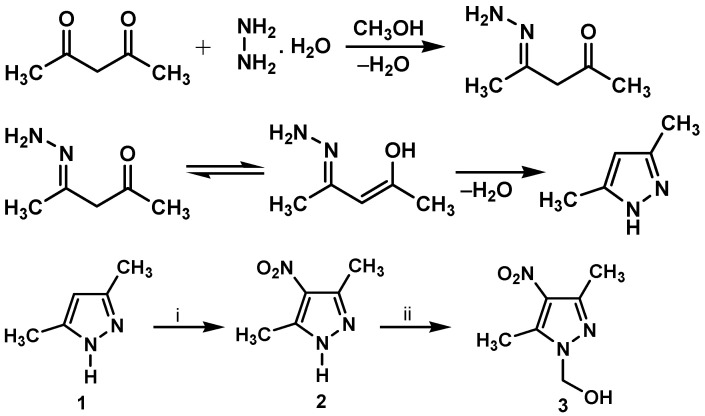
Synthesis of methylolpyrazole derivatives. Reagents: acetylacetone, hydrazine hydrate (**1**); (i) HNO_3_/H_2_SO_4_ (**2**); (ii) CH_2_O, MeOH (**3**).

Pyrazoles **1** and **2** were synthesized in good yields (84% and 59%, respectively) according to previously published methods [39,40,41] (Scheme 1). Synthesis of 1-(hydroxymethyl)-pyrazole derivative **3** was realized in good yields (71%), as described by Dvoretzky and Richter [42,43]. Methylol compound **3** was synthesized for the first time by our group and is not described previously in the literature (Scheme 1) [34,35,43].

Compounds **4a**–**f** and **5a**–**e** were synthesized by the modified Mannich reaction, performed in two steps.

**Scheme 2:**
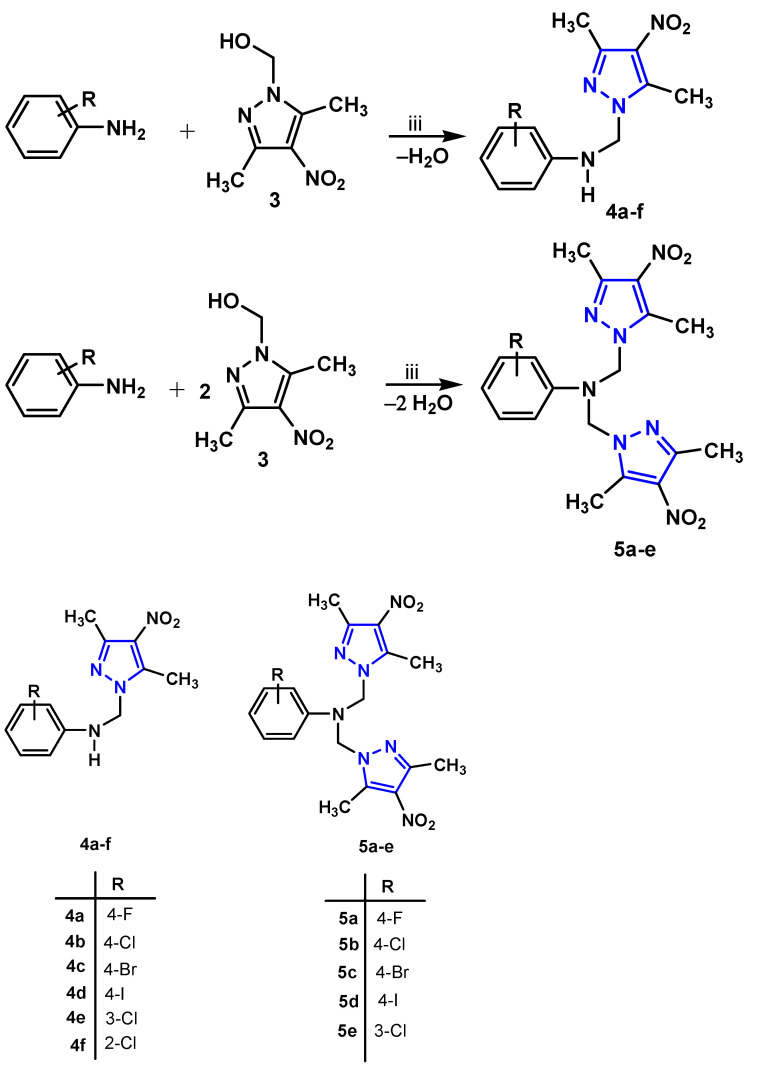
Synthesis of substituted pyrazoles **4a**–**f** and **5a**–**e**. Reagents (iii) CH_2_Cl_2_.

In the first step, pyrazole **2** reacted with a 37% formaldehyde solution, forming the intermediate compound 1-hydroxymethyl-3,5-dimethyl-4-nitropyrazole **3**, and in the second step, depending on the number of moles of the intermediate pyrazole compound **3**, which reacts with halogenated anilines, leads to obtaining two series of halogenoaminopyrazole compounds with one pyrazole nucleus **4a**–**f**, respectively, with two pyrazole nuclei **5a**–**e** (Scheme 2).

The structural formulas of all the synthesized compounds, both the intermediate ones and the final ones, were confirmed by spectroscopic and microanalysis methods, and the values of the R_f_ retention factors were mentioned in the experimental section.

### 2.2. Spectroscopic Characterization of Compounds ***4a***–***f*** and ***5a***–***e***

#### 2.2.1. IR Spectra

The molecular structure of the new compounds **4a**–**f** and **5a**–**e**, respectively, was confirmed by the IR spectra made in the potassium bromide pellet. The absorption bands characteristic of compounds **4a**–**f**, which indicated their secondary amine structure, were found in the range 3396–3124 cm^−1^, and these are attributed to the v_N-H_ stretching vibration frequency. These absorption bands disappeared in compounds **5a**–**e**, being tertiary amines.

The characteristic bands due to the frequency of stretching vibrations v_Carom-N_ at 1217–1213 cm^−1^, respectively, v_Calif-N_ at 1192–1100 cm^−1^ can be observed for all synthesized compounds.

The two characteristic absorption bands of the stretching vibrations ν(NO_2_)asym, respectively ν(NO_2_)sym, located in the range 1563–1491 cm^−1^ and 1361–1304 cm^−1^, respectively, are found in all compounds.

The two absorption bands of the pyrazole nuclei due to the frequency of stretching vibrations were found in the ranges of 1420–1402 cm^−1^ and 1189–1071 cm^−1^, respectively.

#### 2.2.2. Electronic Spectra

The electronic spectra of the new compounds were recorded in an ethanolic solution and showed characteristic values in the range of 246–300 nm for all compounds. Two characteristic bands, in the range of 334–520 nm and 238–323 nm, respectively, were recorded for the chromophores of compounds **4a**–**f**. A characteristic band, in the range 428–564 nm, was recorded for the chromophores of compounds **5a**–**e**. All these bands were assigned to π-π* transitions.

#### 2.2.3. NMR Spectral Analysis

The structures of the new halogenoaminopyrazole compounds with one pyrazole nucleus **4a**–**f**, respectively, and two pyrazole nuclei **5a**–**e** were confirmed by NMR spectroscopy. The structure of the compounds **4a**–**f** was elucidated by the spin coupling between the protons of the methylene group (NHCH_2_) and the proton of the NH group. The protons of the methylene group appear as a singlet in the region 5.34–5.55 ppm. For compounds **5a**–**e** the NH group disappears and the protons of the methylene group appear as a singlet in the region 5.45–5.60 ppm. The pyrazole ring’s hydrogen atoms displayed anticipated chemical shift values, identifying the type of substituents.

The methylene groups are represented by the chemical shifts. In the ^13^C-NMR spectra (δ = 59.3–60.7 ppm) for compounds **4a**–**f**, respectively (δ = 65.1–66.4 ppm) for compounds **5a**–**e**. The least deshielded chemical shifts of the methylene carbon atom belong to compound **4e**, respectively, **5e** in both compounds, and the halogen atom is the disubstituted meta chlorine atom. The most unscreened chemical shifts of the methylene carbon atom belong to compound **4a**, respectively, and **5a** in both compounds, the halogen atom being the fluorine atom in position 4 of the phenyl ring.

The recorded NMR spectra for the compounds are attached in the Supplementary Materials.

#### 2.2.4. X-Ray Crystallography

From the two series, only two compounds formed single crystals. The other compounds did not form suitable crystals for single-crystal X-ray diffraction measurements, even though they were isolated as microcrystalline solids. Summary of crystallographic data and refinement for **4a** and **5d** are presented in Tables S1 and S2 in the Supplementary Materials.

**Figure 4:**
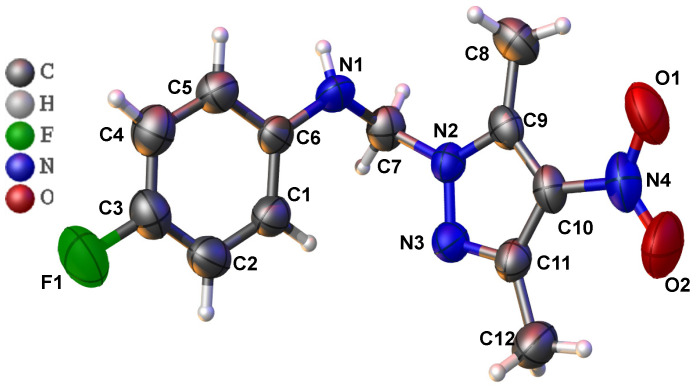
X-ray molecular structure of **4a** with color and atoms numbering scheme. Thermal ellipsoids representation with 50% probability.

So compound **4a** crystallizes in a monoclinic system, space group P2_1_/n (#14). The asymmetric units represented in Figure 4 are shown.

**Figure 5:**
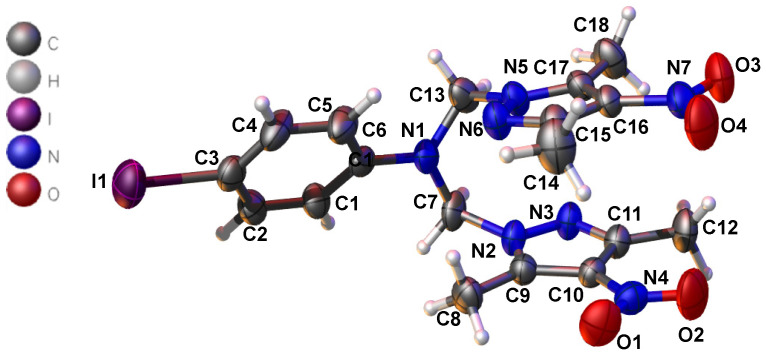
X-ray molecular structure of compound **5d** with color and atom numbering. Thermal ellipsoids representation with 50% probability.

The compound **5d** crystallizes in a triclinic system, space group P-1. The asymmetric units represented in Figure 5 are shown.

**Figure 6:**
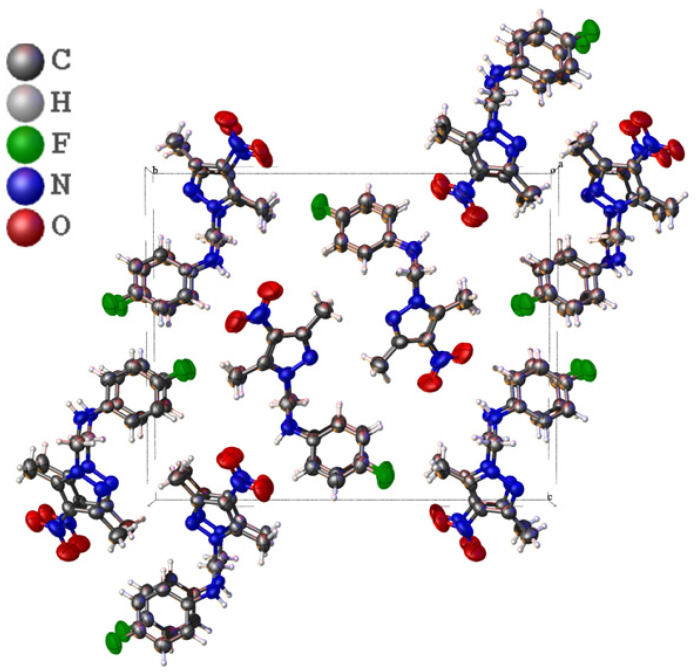
Crystal packing for (**4a**) along *a* axis.

**Figure 7:**
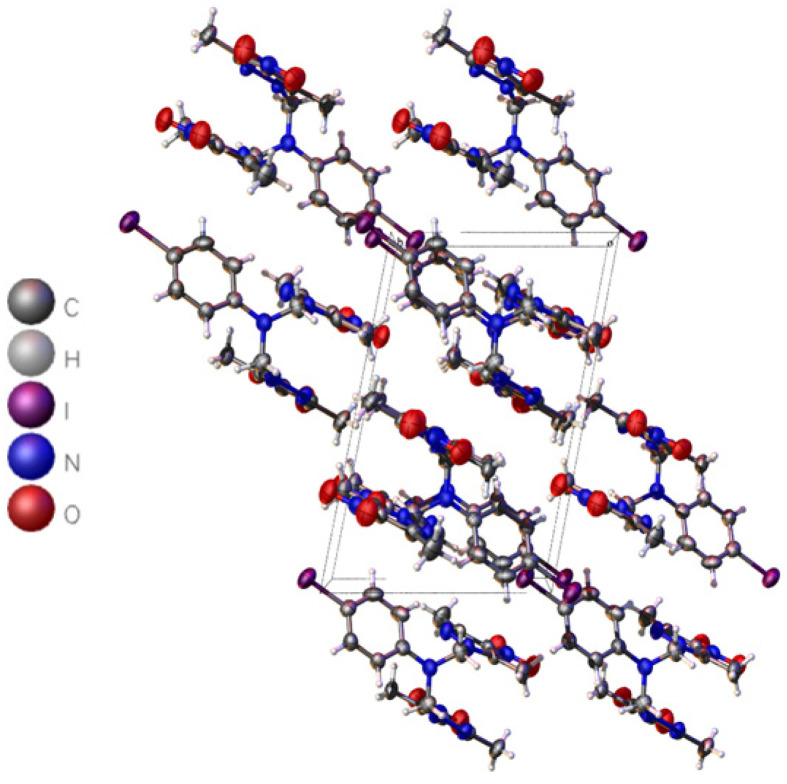
Crystal packing for (**5d**) along *a* axis.

Figure 6 and Figure 7 are selected crystal packings for **4a** and **5d** compounds. They are presented in their entirety in the Supplementary Materials.

### 2.3. Biological Activity

#### 2.3.1. Evaluation of the In Vitro Cytotoxicity of the New Compounds **4a**–**f** and **5a**–**e**

The cytotoxicity of the halogenoaminopyrazole derivatives **4a**–**f** and **5a**–**e** was tested on L929 mouse fibroblast cells, and their anti-tumor activity was evaluated on the human carcinoma epithelial cell line HEp-2. The experiments were performed in a concentration range of 3.1–50 μg/mL.

The MTT test was used, 3-(4,5-dimethylthiazol-2-yl)-2,5-diphenyl tetrazolium bromide), which is reduced to insoluble purple-blue formazan crystals precipitated by living cells. Cell viability assessed using the MTT assay reflects the activity of mitochondrial enzymes in treated cells compared to the control culture of untreated cells.

The results showing the cytotoxicity of the compounds according to the tested concentration and presented as mean ± SD (*n* = 3) are attached in the Supplementary Materials as Tables S3 and S4.

Table 1 shows the IC_50_ results of the cell viability test of compounds from series **4a**–**f** and **5a**–**e** following in vitro testing on normal NCTC fibroblasts and HEp-2 epithelial tumor cells at 48 h.

Regarding in vitro anti-tumor activity testing, the results of MTT assays showed that after 48 h of treatment of HEp-2 human cervical carcinoma cells with the studied compounds, it was found that some compounds from both series (**4a**, **4b**), (**5a**, **5b**, **5d**, **5e**), respectively, showed moderate anti-tumor activity, with IC_50_ values ranging between 26.61 and 47.31 µg/mL.

The other compounds presented IC_50_ values higher than 50 µg/mL, thus not being relevant.

To compare the anti-tumor activity of the synthesized halogenoaminopyrazole derivatives **4a**–**f** and **5a**–**e**, dioscin, a compound with strong anti-tumor activity, was used, which was tested under the same conditions on HEp-2 cells by MTT assays.

Dioscin, the reference compound, showed strong antitumor activity with a value of IC_50_ of 14.65 µg/mL.

Compound **5d**, which contains the iodine atom in position 4 of the phenyl ring, shows the best anti-tumor activity at an IC_50_ value of 26.61 µg/mL compared to the compounds studied from the two series, but moderately compared to dioscin, the compound of reference.

#### 2.3.2. Antimicrobial Activity

**Figure 8:**
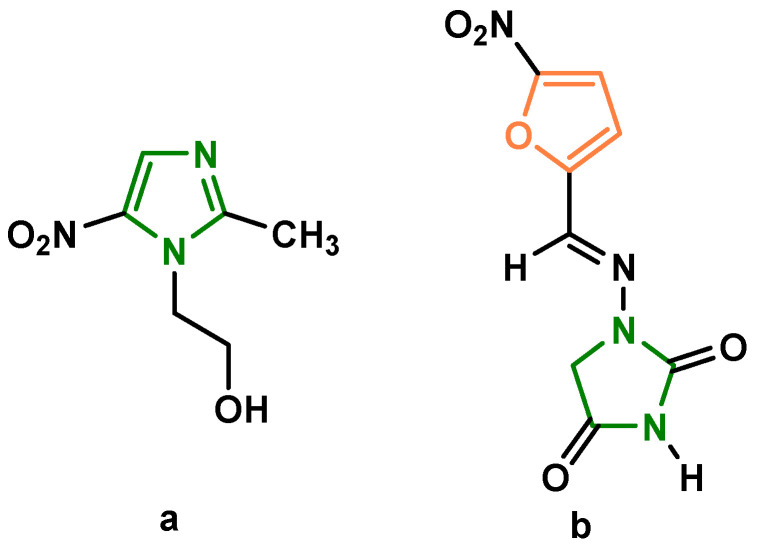
Molecular structures of reference drugs (**a**) metronidazole, (**b**) nitrofurntoin.

The antibacterial activity against planktonic microbial cells of the two series of newly synthesized compounds **4a**–**f** and **5a**–**e** was screened on four bacterial strains, namely, two Gram-positive strains, *Staphylococcus aureus* ATCC25923, and *Enterococcus faecalis* ATCC29212, and two Gram-negative strains, *Pseudomonas aeruginosa* ATCC27853, and *Escherichia coli* ATCC25922. Two reference drugs, commercial formulations Arena (tablets), metronidazole (a), and nitrofurantoin (b), were used to compare the antibacterial activity of the synthesized compounds, whose structural formulas are shown in Figure 8. 

We chose the two reference drugs, which are structurally similar, having nitro groups on the heterocyclic ring. In addition, the synthesized pyrazole compounds are azoles, and the reference drugs are azoles or azole derivatives.

##### Qualitative Antibacterial Analysis

The qualitative results of the analysis of the antimicrobial activity of the newly synthesized compounds **4a**–**f** and **5a**–**e** showed the presence of the inhibitory effect on microbial growth, indicated by the appearance of growth inhibition zones, and are presented in Table 2.

More than half of the compounds, namely, six of eleven screened for the qualitative evaluation of the antimicrobial activity, produced a large zone of inhibition. Chlorine-containing compounds in the “4” position of the phenyl ring, **4b** and **5b**, inhibited the growth of all tested bacteria. Fluorine-containing compounds in the “4” position of the phenyl ring, **4a** and **5a**, were noted as also inhibiting the growth of bacteria, but only on three bacterial strains tested. Chlorine-containing compounds in the “2” (**4f**) and “3” (**4e**) positions of the phenyl ring inhibited the growth of bacteria on three bacterial strains among the four tested. Compounds **4c**, **4d**, **5c**, **5d**, and **5e** did not inhibit the growth of bacteria on any of the strains tested.

##### Quantitative Antibacterial Analysis

After testing the antibacterial activity of the synthesized compounds, the MIC expressed in μg/mL was established and quantified in Table 3.

Among the 11 compounds synthesized after testing the antibacterial activity, only 6 compounds were the most active against *Staphylococcus aureus* ATCC25923 compared to the two reference drugs, metronidazole and nitrofurantoin. Compound **4a**, which has the fluorine atom in position “4” of the phenyl ring, showed the best activity against *Staphylococcus aureus* ATCC25923, with a MIC of 230 μg/mL compared to reference drugs (metronidazole, nitrofurantoin). Also, compounds **4b**, **4e**, and **4f**, which have chlorine in positions “4”, “3”, and “2” of the phenyl ring with a pyrazole nucleus, and **5a** and **5b** and a fluorine atom in positions “4” of the phenyl ring, respectively, and a chlorine atom in the position “4” of the phenyl ring with 2 pyrazole nuclei showed good activity against the same bacterial strain, with a MIC of 460 μg/mL, much lower than those obtained for the reference drugs (metronidazole, nitrofurantoin). Against the *Enterococcus faecalis* ATCC29212 bacterial strain, only two compounds, **4b** and **5b**, are the most active. The compounds **4b** and **5b** share the grafted chlorine atom in position “4” of the phenyl nucleus but differ in the number of pyrazole nuclei. Against *Pseudomonas aeruginosa* ATCC27853, 4 compounds are the most active **4b**, **4f**, **5a**, and **5b**, these compounds contain either the fluorine or chlorine atoms of the phenyl nucleus. Against *Escherichia coli* ATCC25922 5 compounds are the most active, the same compounds **4b**, **4e**, **4f**, **5a**, and **5b**, with an MIC of 460 μg/mL compared to reference drugs. Only compound **5c**, which has the bromine atom in position “4” of the phenyl ring, with two pyrazole nuclei, is the least active compound against all 4 bacterial strains with an MIC of 3750 μg/mL.

##### Quantitative Antibiofilm Analysis 

The minimum inhibitory concentrations of bacterial biofilms (MBIC) in μg/mL for the tested compounds in series **4a**–**f** and **5a**–**e** were measured to determine their antibiofilm activity (Table 4).

The synthesized compounds **4a**–**f** and **5a**–**e** exhibited excellent antibiofilm effectiveness against the *Staphylococcus aureus* ATCC25923 strain in comparison to the standard drugs. Compounds **4a** and **5a** showed high activity, inhibiting a biofilm with an MBIC of 230 µg/mL against the bacteria strain, which is significantly lower than metronidazole and nitrofurantoin. They contain the fluorine atom. After them follows the compounds **4b**, **4e**, **4f**, and **5b** having an MBIC of 460 µg/mL. They contain the chlorine atom. No compound showed antibiofilm activity against *Enterococcus faecalis* ATCC29212 bacterial strain, compared to the reference drugs. Five compounds are the most active against the *Pseudomonas aeruginosa* ATCC27853 strain, namely, **4b**, **4d**, **4f**, **5a**, and **5b**, with an MBIC of 460 µg/mL, compared to reference drugs. Compound **4d** contains the iodine atom in position “4” of the phenyl nucleus and has good anti-biofilm activity against *Staphylococcus aureus* strain ATCC25923 compared to the reference drugs. Only three compounds, **4f**, **5a**, and **5b**, showed good antibiofilm activity against the *Escherichia coli* ATCC25922 bacterial strain, with an MBIC of 460 µg/mL, compared to reference drugs. Compound **5c**, with the bromine atom in position “4” of the phenyl ring, and two pyrazole nuclei, is the least active against the three bacterial strains *Enterococcus faecalis* ATCC29212, *Pseudomonas aeruginosa* ATCC27853, *Escherichia coli* ATCC25922 with a MIC of 1870 μg/mL, 1870 μg/mL, and 3750 μg/mL, respectively, compared to reference drugs, but better than nitrofurantoin against *Staphylococcus aureus* ATCC25923.

We can conclude that the results of the antimicrobial activity show us that the most promising synthesized compounds **4b** and **5b** could be considered antibacterial with a broad spectrum of action, and the best compounds, **4a** and **5a**, could be considered antibiofilm agents against *Staphylococcus aureus* ATCC25923.

## 3. Materials and Methods

### 3.1. General Chemical Characterization Techniques

The purity of the synthesized compounds was evidenced by determining the technical melting temperature TLC, Merck plates with silica gel, elution system *n*-butanol:acetic acid:water = 4:1:5 *v*/*v*/*v*, spot detection with UV Lamp (λ = 254 nm and 365 nm), by elemental analysis (Perkin Elmer 2400 Series II CHNS/O), (Perkin ElmerWaltham, MA, USA) the experimental error being within ±0.4%. IR spectra were recorded with a Varian Resolutions spectrometer, and electronic ones were recorded with a VSU-2P Zeiss-Jena spectrophotometer (Analytik Jena GmbH, Jena, Germany). ^1^H-NMR and ^13^C-NMR spectra were recorded with a Varian Gemini 300BB spectrometer (Systems, Palo Alto, CA, USA) at 300 MHz for ^1^H and 75 MHz for ^13^C in CDCl_3_ with TMS as an internal standard.

X-ray crystallography. Crystal data were collected on a Rigaku RAXIS RAPID II diffractometer (Rigaku, Malvern, UK) using graphite monochromatic Mo-Kα radiation (λ = 0.71075 A) and the ω-ϕ scanning technique at room temperature. The single crystals were mounted on fiberglass. Data were collected with the Crystal Clear program. The structures were solved by direct methods [44] and anisotropically refined using a full-matrix least-squares application based on F2. Atoms were refined, and calculations were performed using Olex2 [45]. Details of crystal parameters, data collection, and refinement for compounds **4a** and **5d** are listed in Table S1. A summary of selected bond lengths (Å) and angles (o) is shown in Table S2. CCDC 2359642-2359643, include all additional crystallographic data of compounds **4a** and **5d**.

### 3.2. General Method for the Synthesis of Series Compounds ***4a***–***f*** and ***5a***–***e***

Synthesis of compound **4a**

A 5 mL solution of 4-fluoroaniline (5.8 mmol) in methylene chloride was added dropwise to a solution of the corresponding 1-(hydroxymethyl)-3,5-dimethyl-4-nitropyrazole **3** (5.8 mmol) in 20 mL methylene chloride. The crystallized crude product was obtained after stirring the reaction mixture for 30 h and removing the solvent under vacuum, monitoring the progress of the reaction by thin layer chromatography.

Synthesis of *N-[(3,5-dimetyl-4-nitro-1H-pyrazol-1-yl)-methyl]-4-fluoroaniline* (**4a**)

Yield 55%; mp 117–119 °C; R_f_ 0.14; ^1^H-NMR (300 MHz, CDCl_3_) δ: 2.51, 2.63 (2s, 6H, 2Me), 5.40 (bs, 1H NH), 5.53 (s, 2H, CH_2_N), 6.85–6.98 (m, 4H, ^2^*J*_C–F_ = 22.6 Hz, H-2′, H-3′, H-5′, H-6′); ^13^C-NMR (75 MHz, CDCl_3_) δ: 11.7, 14.3 (2CH_3_),60.7 (CH_2_N),116.1, 116.2 (4C, C-2′, C-3′, C-5′, C-6′), 131.6 (C-4), 140.2, 140.9, 146.7 (C-3, C-5, C-1′).

IR (KBr, cm^−1^) ν 3418 w (NH), 1216 vi (C_aliphatic_-N), 1318 m (C_aromatic_-N), 1563 i (NO_2_as), 1346 vi (NO_2sim_), 1402 w, 1122 w (pyrazole ring); UV-Vis. *λ_max_* (log ε) 330 (1.30) nm; 520 (0.21); Anal. calcd. for C_12_H_13_FN_4_O_2_ (264.26): C 54.54; H 4.96; N 21.20 Found: C 54.92; H 4.79; N 20.83.

Synthesis of *N-[(3,5-dimetyl-4-nitro-1H-pyrazol-1-yl)-methyl]-4-chloroaniline* (**4b**)

Compound **4b** was synthesized according to the general procedure described for **4a**.

Yield 80%; mp 99–100 °C; R_f_ 0.76; ^1^H-NMR (300 MHz, CDCl_3_) δ: 2.50, 2.68 (2s, 6H, 2Me), 5.01 (bs, 1H, NH),5.42 (s, 2H, CH_2_N), 6.76 (d, 2H, *J* = 8.5 Hz, H-2′, H-6′), 7.15 (d, 2H, *J* = 8.5 Hz, H-3′, H-5′); ^13^C-NMR (75 MHz, CDCl_3_) δ: 11.7, 14.3 (2CH_3_),59.7 (CH_2_N), 115.5, 129.3, (4C, C-2′, C-3′, C-5′, C-6′), 124.8 (C-4′), 131.5 (C-4), 140.2, 143.3, 146.1 (C-1′, C-3, C-5). IR (KBr, cm^−1^) ν 3281 m (NH), 1213 i (C_aliphatic_-N), 1274 i (C_aromatic_-N), 1560 i (NO_2_as), 1357 vi (NO_2sim_), 1400 w, 1155 w (pyrazole ring); UV-Vis. *λ_max_* (log ε) 295 (1.21); 505 (0.20) nm; Anal. calcd. for C_12_H_13_ClN_4_O_2_ (280.71): C 51.34; H 4.67; N 19.96 Found: C 50.94; H 4.89; N 19.63.

Synthesis of *N-[(3,5-dimetyl-4-nitro-1H-pyrazol-1-yl)-methyl]-4-bromoaniline* (**4c**)

Compound **4c** was synthesized according to the general procedure described for **4a**.

Yield 86%; mp 118–120 °C; R_f_ 0.80; ^1^H-NMR (300 MHz, CDCl_3_) δ: 2.42, 2.60 (2s, 6H, 2Me),4.80(bs, 1H, NH),5.34 (d, 2H, *J* = 4.7 Hz, CH_2_N), 6.63 (d, 2H, *J* = 8.8 Hz, H-2′, H-6′), 7.21 (d, 2H, *J* = 8.8 Hz, H-3′, H-5′); ^13^C-NMR (75 MHz, CDCl_3_) δ: 11.8, 14.3 (2CH_3_), 59.6 (CH_2_N), 112.2 (C-4′), 116.1, 132.4 (4C, C-2′, C-3′, C-5′, C-6′), 133.1 (C-4), 143.9 (C-1′), 140.7, 144.4, 146.7 (C-1′, C-3, C-5). IR (KBr, cm^−1^) ν 3396 vi (NH), 1216 i (C_aliphatic_-N), 1319 m (C_aromatic_-N), 1561 i (NO_2_as), 1356 vi (NO_2sim_), 1402 m, 1162 m (pyrazole ring); UV-Vis. *λ_max_* (log ε) 281 (1.20), 334 (1.21) nm; Anal. calcd. for C_12_H_13_BrN_4_O_2_ (325.16): C 44.33; H 4.03; N 17.23 Found: C 44.72; H 4.22; N 16.98.

Synthesis of *N-[(3,5-dimetyl-4-nitro-1H-pyrazol-1-yl)-methyl]-4-iodoaniline* (**4d**)

Compound **4d** was synthesized according to the general procedure described for **4a**.

Yield 76%; mp 91–92 °C; R_f_ 0.78; ^1^H-NMR (300 MHz, CDCl_3_) δ: 2.42, 2.61 (2s, 6H, 2Me), 4.91 (t, 1H, *J* = 4.7 Hz, NH), 5.34 (d, 2H, *J* = 4.7Hz, CH_2_N), 6.53 (d, 2H, *J* = 8.8 Hz, H-2′, H-6′), 7.39 (d, 2H, *J* = 8.8 Hz, H-3′, H-5′); ^13^C-NMR (75 MHz, CDCl_3_) δ: 11.8, 14.4 (2CH_3_), 59.4 (CH_2_N), 81.7 (C-I; C-4), 116.5, 138.2 (4C, C-2′, C-3′, C-5′, C-6′), 140.2, 144.5, 146.3 (C-1′, C-3, C-5). IR (KBr, cm^−1^) ν 3124 m (NH), 1175 i (C_aliphatic_-N), 1300 m (C_aromatic_-N), 1599 vi (NO_2_as), 1304 vi (NO_2sim_), 1415 m, 1003 w (pyrazole ring); UV-Vis. *λ_max_* (log ε) 246 (1.20), 351 (1.21) nm; Anal. calcd. for C_12_H_13_IN_4_O_2_ (372.17): C 38.73; H 3.52; N 15.05 Found: C 38.95; H 3.70; N 15.26.

Synthesis of *N-[(3,5-dimetyl-4-nitro-1H-pyrazol-1-yl)-methyl]-3-chloroaniline* (**4e**)

Compound **4e** was synthesized according to the general procedure described for **4a**.

Yield 84%; mp 110–111 °C; R_f_ 0.78; ^1^H-NMR (300 MHz, CDCl_3_) δ: 2.49, 2.68 (2s, 6H, 2Me), 5.08 (t 1H, *J* = 6.3 Hz, NH), 5.41 (d, 2H, *J* = 6.3 Hz, CH_2_N), 6.67–6.70 (m, 1H, H-6′), 6.75–6.81 (m, 2H, H-2′, H-4′), 7.10 (t, 1H, *J* = 8.0 Hz, H-5′); ^13^C-NMR (75 MHz, CDCl_3_) δ: 11.7, 14.3 (2CH_3_),59.3(NCH_2_) 112.3, 114.1, 119.9, 130.5 (4C, C-2′, C-3′, C-5′, C-6′), 131.5 (C-4), 135.1 (C-3′), 140.3, 145.9, 146.2 (C-3, C-5, C-1′). IR (KBr, cm^−1^) ν 3388 m (NH), 1214 i (C_aliphatic_-N), 1315 m (C_aromatic_-N), 1561 i (NO_2_as), 1353 vi (NO_2sim_), 1412 m, 1156 w (pyrazole ring); UV-Vis. *λ_max_* (log ε) 300 (1.21); 515 (0.20) nm; Anal. calcd. for C_12_H_13_ClN_4_O_2_ (280.71): C 51.34; H 4.67; N 19.96 Found: C 50.99; H 4.86; N 19.77.

Synthesis of *N-[(3,5-dimetyl-4-nitro-1H-pyrazol-1-yl)-methyl]-2-chloroaniline* (**4f**)

Compound **4f** was synthesized according to the general procedure described for **4a**.

Yield 84%; mp 134–135 °C; R_f_ 0.10; ^1^H-NMR (300 MHz, CDCl_3_) δ: 2.52, 2.72 (2s, 6H, 2Me), 5.30(bs, 1H, NH), 5.55 (s, 2H, CH_2_N), 6.79 (t, 1H, *J* = 8.0 Hz, H-4′), 7.13 (d, 1H, *J* = 8.0 Hz, H-3′), 7.21 (t, 1H, *J* =8.0 Hz, H-5′), 7.28 (d, 1H, *J* = 8.0 Hz, H-6′); ^13^C-NMR (75 MHz, CDCl_3_) δ: 11.7, 14.3 (2CH_3_); 59.5 (CH_2_N), 113.4, 120.3, 128.1, 128.4 (4C, C-3′, C-4′, C-5′, C-6′), 131.0 (C-4), 140.3, 140.9, 146.0 (C-1′, C-3, C-5). IR (KBr, cm^−1^) ν 3390 i (NH), 1214 vi (C_aliphatic_-N), 1318 m (C_aromatic_-N), 1563 i (NO_2_as), 1346 vi (NO_2sim_), 1420 w, 1136w (pyrazole ring); UV-Vis. *λ_max_* (log ε) 300 (1.03) nm; 510 (0.19); Anal. calcd. for C_12_H_13_ClN_4_O_2_ (280.71): C 51.34; H 4.67; N 19.96 Found: C 51.62; H 4.49; N 20.23.

Synthesis of compound **5a**

5 mL of chloromethylene solution of 4-fluoroaniline (5.8 mmol) was dropped over a corresponding chloromethylene solution of 1-(hydroxymethyl)-3,5-dimethyl-4-nitro pyrazole **3** (11.6 mmol). The crude product **5a** nicely crystallized after stirring the reaction mixture for 30 h and removing the solvent under vacuum, monitoring the progress of the reaction by thin layer chromatography.

Synthesis of *N,N-bis-[(3,5-dimetyl-4-nitro-1H-pyrazol-1-yl)-methyl]-4-fluoroaniline* (**5a**)

Yield 95%; mp 78–80 °C; R_f_ 0.72 ^1^H-NMR (300 MHz, CDCl_3_) δ: 2.29, 2.41 (2s, 6H, 2Me), 5.45 (s, 4H, 2NCH_2_N), 6.85–6.98 (m, 4H, H-2′, H-3′, H-5′, H-6′); ^13^C-NMR (75 MHz, CDCl_3_) δ: 11.4, 14.3 (2CH_3_), 66.4 (2NCH_2_N); 116.8 (d, ^2^*J*_C–F_ = 22.6 Hz, C-3′, C-5′), 125.3 (d, ^3^*J*_C–F_ = 8.3 Hz, C-2′, C-6′), 131.5 (C-4), 141.2, 146.6 (C-1′, C-3, C-5), 162.0 (d, ^1^*J*_C-F_ = 246.2 Hz, C-4′).

IR (KBr, cm^−1^) ν 1217 i (C_aliphatic_-N), 1316 i (C_aromatic_-N), 1560 vi (NO_2_as), 1300 vi (NO_2sim_), 1406 i, 1192 m (pyrazole ring); UV-Vis. *λ_max_* (log ε) 281 (1.20), 331 (1.30) nm; Anal. calcd. for C_18_H_20_FN_7_O_4_ (417.40):C 51.80; H 4.83; N 23.49; Found: C 51.54; H 4.68; N 23.69.

Synthesis of *N,N-bis-[(3,5-dimetyl-4-nitro-1H-pyrazol-1-yl)-methyl]-4-chloroaniline* (**5b**)

Compound **5b** was synthesized according to the general procedure described for **5a**.

Yield 79%; mp 132-134 °C; R_f_ 0.33; ^1^H-NMR (300 MHz, CDCl_3_) δ: 2.38, 2.41 (2s, 6H, 2Me), 5.50 (s, 4H, 2NCH_2_N), 6.91 (d, 2H, *J* = 8.5 Hz, H-2′, H-6′), 7.24 (d, 2H, *J* = 8.5 Hz, H-3′, H-5′); ^13^C-NMR (75 MHz, CDCl_3_) δ: 11.6, 14.3 (2CH_3_),65.6 (2NCH_2_N), 123.1, 130.1 (4C, C-2′, C-3′, C-5′, C-6′), 130.3 (C-4′), 13.7 (C-4),143.9 (C-1′),140.7, 143.9,146.7 (C-1′, C-3, C-5). IR (KBr, cm^−1^) ν 1215 i (C_aliphatic_-N), 1374 vi (C_aromatic_-N), 1562 vi (NO_2_as), 1360 vi (NO_2sim_), 1404 m, 1189 m (pyrazole ring); UV-Vis. *λ_max_* (log ε) 323 (1.21), 564 (0.20) nm; Anal. calcd. for C_18_H_20_ClN_7_O_4_ (433.85): C 49.83; H 4.65; N 22.60 Found: C 49.94; H 4.49; N 22.49.

Synthesis of *N,N-bis-[(3,5-dimetyl-4-nitro-1H-pyrazol-1-yl)-methyl]-4-bromoaniline* (**5c**)

Compound **5c** was synthesized according to the general procedure described for **5a**.

Yield 87%; mp 133–135 °C; R_f_ 0.82; ^1^H-NMR (300 MHz, CDCl_3_) δ: 2.46, 2.47 (2s, 6H, 2Me), 5.60 (s, 4H, 2NCH_2_N), 6.93 (d, 2H, *J* = 8.8 Hz, H-2′, H-6′), 7.44 (d, 2H, *J* = 8.8 Hz, H-3′, H-5′); ^13^C-NMR (75 MHz, CDCl_3_) δ: 11.6, 14.3 (2CH_3_), 65.5 (2NCH_2_N), 117.8 (C-4′), 123.2, 133.0 (4C, C-2′, C-3′, C-5′, C-6′), 131.7 (C-4), 140.7, 144.4, 146.7 (C-1′, C-3, C-5). IR (KBr, cm^−1^) ν 1213 i (C_aliphatic_-N), 1313 m (C_aromatic_-N), 1561 vi (NO_2_as), 1360 vi(NO_2sim_), 1401 m, 1147 m (pyrazole ring); UV-Vis. *λ_max_* (log ε) 238 (1.30), 321 (1.40) nm; Anal. calcd. for C_18_H_20_BrN_7_O_4_ (478.30): C 45.20; H 4.21; N 20.50; Found: C 44.81; H 4.02; N 20.31.

Synthesis of *N,N-bis-[(3,5-dimetyl-4-nitro-1H-pyrazol-1-yl)-methyl]-4-iodoaniline* (**5d**)

Compound **5d** was synthesized according to the general procedure described for **5a**.

Yield 86%; mp 158–160 °C; R_f_ 0.71; ^1^H-NMR (300 MHz, CDCl_3_) δ: 2.46, 2.47 (2s, 6H, 2Me), 5.59 (s, 4H, 2NCH_2_N),6.82 (d, 2H, *J* = 8.5 Hz, H-2′, H-6′), 7.61 (d, 2H, *J* = 8.5 Hz, H-3′, H-5′); ^13^C-NMR (75 MHz, CDCl_3_) δ: 11.6, 14.3 (2CH_3_), 65.3 (2NCH_2_N), 87.9 (C-I C-4′), 123.0, 138.9 (4C, C-2′, C-3′, C-5′, C-6′), 131.6 (C-4) 140.7, 145.1, 146.7 (C-1′, C-3, C-5). IR (KBr, cm^−1^) ν 1217 i (C_aliphatic_-N), 1315 m (C_aromatic_-N), 1565 i (NO_2_as), 1359 vi (NO_2sim_), 1401 m, 1189 m (pyrazole ring); UV-Vis. *λ_max_* (log ε) 320 (1.30), 428 (0.48) nm; Anal. calcd. for C_18_H_20_IN_7_O_4_ (525.30): C 41.16; H 3.84; N 18.66; Found: C 41.31; H 3.73; N 18.47.

Synthesis of *N,N-bis-[(3,5-dimetyl-4-nitro-1H-pyrazol-1-yl)-methyl]-3-chloroaniline* (**5e**)

Compound **5e** was synthesized according to the general procedure described for **5a**.

Yield 17%; mp 154–155 °C; R_f_ 0.53; ^1^H-NMR (300 MHz, CDCl_3_) δ: 2.41, 2.44 (2s, 6H, 2Me), 5.54 (s, 4H, 2NCH_2_N), 6.48–6.88 (m, 1H, H-6′), 7.00–7.03 (m, 2H, H-4′, H-6′), 7.16–7.21 (m, 1H, H-5′); ^13^C-NMR (75 MHz, CDCl_3_) δ: 11.7, 14.3 (2CH_3_), 65.1 (2NCH_2_N), 118.6, 121.0, 124.4, 131.0 (4C, C-2′, C-4′, C-5′, C-6′), 131.7 (C-4), 135.2 (C-3′), 140.2, 140.8, 146.8 (C-1′, C-3, C-5). IR (KBr, cm^−1^) ν 1216 i (C_aliphatic_-N), 1313 i (C_aromatic_-N), 1562 vi (NO_2_as), 1357 vi (NO_2sim_), 1402 i, 1151 w (pyrazole ring); UV-Vis. *λ_max_* (log ε) 287 (1.09), 306 (1.03) nm; Anal. calcd. for C_18_H_20_ClN_7_O_4_ (433.85): C 49.83; H 4.65; N 22.60; Found: C 49.55; H 4.81; N 22.43.

### 3.3. Biological Tests

#### 3.3.1. Cytotoxicity of Samples

Merck chemicals were utilized. To assess the biocompatibility of the substances, a stable mouse fibroblast cell line, NCTC (clone 929), was grown in minimal essential medium (MEM) with 10% fetal bovine serum (FBS) and 2 mM L-glutamine, along with 100 U/mL penicillin, 100 µg/mL streptomycin, and 500 µg/mL neomycin. The concentration of the compounds used was 0.06 g/2 mL DMSO. The reference compound used was dioscin (Merck-Darmstat, Germany), which was tested under the same conditions as the studied compounds.

In cytotoxicity experiments, compound solutions were utilized at concentrations of 3.1 μg/mL, 6.25 μg/mL, 12.5 μg/mL, 25 μg/mL, and 50 μg/mL. The NCTC cell suspension was placed into 96-well culture plates with a concentration of 4 × 10^4^ cells/mL and incubated at 37 °C for 24 h in a 5% CO_2_ humidified atmosphere. The compounds under study were placed in sets of three at varying concentrations ranging from 3.1 μg/mL to 50 μg/mL in every well, and the plates were then kept under standard conditions for 48 h. Cell viability was determined through the utilization of the MTT assay. Next, the MTT solution was removed, then isopropanol was added to solubilize the formazan crystals. The colored solution was then measured for absorbance at 570 nm using a Berthold Technologies, LB 940 microplate reader (Bad Wildbad, Germany). The outcomes were determined based on Formula (1) while considering that the recorded optical density is directly related to the number of viable cells in the cell culture being examined [35,36].

% cell viability = (OD sample/OD control) × 100%(1)

Untreated cells were used as control, considered 100% viable cells.

For each tested compound, a graph representing percentage cell viability versus concentrations (in the range 3.1–50 µg/mL) was obtained. Then, the IC_50_ value was calculated by using ED_50_ plus V1.0 software.

#### 3.3.2. Qualitative Evaluation of the Antimicrobial Activity

For testing the antimicrobial/antibiofilm activity, the concentration of the studied compounds was identical to 0.06 g/2 mL DMSO. 

0.5 Standard McFarland suspension was used, which was inoculated onto Mueller Hinton agar plates, (Remel, Lenexa, KS, USA), 5 µL of each compound was spotted and incubated overnight at 37 °C. Antimicrobial activity was observed as zones of inhibition formed around the spot [34,35].

#### 3.3.3. Testing the Antimicrobial Activity on Bacterial Strains Using Quantitative Methods

The minimum inhibitory concentrations (MIC) were determined by the serial microdilution method in a liquid medium (Tryptone Soy Broth), on 96-well plates. Serial binary dilutions of the compound stock solution (10 mg/mL in DMSO) were carried out in 90 µL of culture medium and 10 µL of 10^6^ CFU/mL (Colony Forming Units) microbial suspension. Each test included a positive control (wells with culture medium and microbial suspension) and a negative control for medium sterility. After incubation at 37 °C for 24 h the plates were visually examined, and optical density was measured at 620 nm. Compulsory sterilization monitoring revealed the absence of bacterial growth, with the liquid content retaining its clarity and transparency. The MIC value (µg/mL) for the chemical compound was determined by the concentration in the final well without bacterial growth [34,35].

All experiments were performed in triplicate.

#### 3.3.4. Antibiofilm Assay

Microorganisms were grown in 96-well plates filled with nutrient broth along with test substances, followed by incubation at 37 °C for 24 h. Plates were cleaned and rinsed thrice using SPW solution (sterile physiological water). After that, the attached cells were treated with 110 μL of methanol for 5 min, and then the methanol was removed by turning it upside down. After the cells adhered, they were stained with 1% alkaline crystal violet solution (110 μL/well) for 15 min. Once stained, the solution was removed, and the plates were washed with running tap water. The biofilms created by microbes on plastic plates were mixed in 33% acetic acid through bubbling, and the strength of the colored mixture was measured by examining the absorbance at 492 nm [34,35].

The antimicrobial/antibiofilm activity of the compounds was evaluated against two antibiotics, commercial formulations of metronidazole and nitrofurantoin.

### 3.4. Statistical Analysis

Student’s *t*-test was utilized to conduct a statistical analysis of the data. The data were presented as average ± standard deviation for three separate samples (*n* = 3). Statistical significance was determined at *p* < 0.05 [35,36].

## 4. Conclusions

In the presented work, the structures of the newly synthesized compounds **4a**–**f** and **5a**–**e** were confirmed by the assignment of NMR, FTIR, UV-VIS, and elemental analysis spectra. From the two series, compounds **4a** and **5d** formed single crystals and were characterized by X-ray diffraction.

The cell viability results were dependent on the tested concentration, so the in vitro cytotoxicity evaluation of the newly synthesized compounds showed their biocompatibility with normal NCTC fibroblasts up to a concentration of 12.5 μg/mL.

The best cell viability on NCTC at a concentration of 6.25 μg/mL at 48 h, they presented compound **4a**, with a cell viability value of 110.87%, and, respectively, compound **5d**, of 103.10%.

Of the two series of compounds studied, compound **5d** shows good antitumor activity with an IC_50_ value of 26.61 μg/mL on HEp-2 cells at 48 h.

The results obtained following the evaluation and antimicrobial testing of the newly synthesized compounds showed that they can be antimicrobial agents.

Six noted compounds, fluorine-containing compounds on the phenyl ring, **4a** and **5a,** and chlorine-containing compounds on the phenyl ring, **4b**, **4e**, **4f**, and **5b**, were proved to be the most active compared to the two reference drugs, metronidazole and nitrofurantoin.

Compound **4b**, the most active compound, which contains the chlorine atom in the “4” position of the phenyl nucleus, has a broad spectrum of action against the four tested bacterial strains, with a MIC value of 460 μg/mL.

All the synthesized compounds show very good antibiofilm activity against the bacterial strain *Staphylococcus aureus* ATCC25923 compared to the two reference drugs.

Compounds **4a** and **5a**, which have the fluorine atom, have μg/mL of the bacterial strain *Staphylococcus aureus* ATCC25923 with an MBIC of 230 μg/mL.

Chlorine-containing compounds, **4b**, **4e**, **4f**, and **5b**, are the most active against *Enterococcus faecalis* ATCC29212, *Pseudomonas aeruginosa* ATCC27853, and *Escherichia coli* ATCC25922, with MBIC of 460 μg/mL.

Fluorine-containing compound **4a**, which crystallizes in the monoclinic system, has the best antibacterial activity against *Staphylococcus aureus* ATCC25923, with a MIC of 230 μg/mL and moderate antitumor activity. Compounds **4a** and **5a** containing fluorine, with a higher polarity of the molecule, offer a better interaction with the hydrophilic receptor, and a more pronounced antimicrobial activity.

Iodine-containing compound **5d**, which crystallizes in the triclinic system, induced apoptosis of HEp-2 cervical carcinoma cells with a value of 20.84% at a concentration of 50 μg/mL. Also, this compound exhibited good biocompatibility when tested on normal fibroblasts over the entire concentration range. Iodine-containing Mannich base **5d** has the best antitumor activity due to the presence of iodine on the molecule, which gives it a weaker hydrophilic character and a better interaction with the receptor.

The obtained results suggest ed that compound **5d** could be a promising therapeutic agent in cervical cancer therapy.

To correlate the relationship between structure and biological activity, the results showed that compounds **4a** and **5a** with fluorine atoms have the best antibacterial and anti-biofilm activity, while compound **5d**, with an iodine atom, has the best IC_50_ value of the anti-tumoral activity

In general, synthesized Mannich bases exhibit strong antimicrobial properties and can effectively prevent biofilm formation, making them promising options for therapeutic use.

## Figures and Tables

**Table 1:** IC_50_ values resulting from the cell viability test in series **4a**–**f** and **5a**–**e** tested in vitro on NCTC normal fibroblasts and HEp-2 epithelial tumor cells at 48 h of treatment.

Sample	Structure of the Synthesized Compounds	IC_50_ µg/mL on NCTC Cells 48 h	IC_50_ µg/mL on HEp-2 Cells 48 h
**4a**	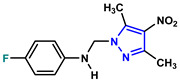	46.42 ± 1.33	40.21 ± 1.26
**4b**	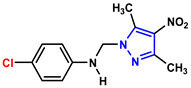	36.11 ± 1.23	47.31 ± 3.38
**5a**	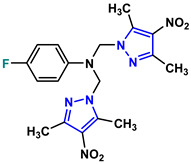	>50	40.53 ± 0.79
**5b**	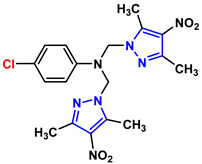	29.81 ± 0.32	>50
**5d**	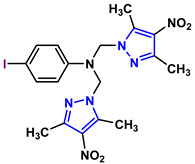	>50	26.61 ± 0.11
**5e**	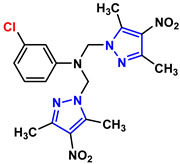	20.30 ± 0.45	31.73 ± 0.48
dioscin		15.78 ± 2.19	14.65 ± 2.07

**Table 2:** Qualitative evaluation of the antimicrobial activity of the synthesized compounds of the two series **4a**–**f** and **5a**–**e**.

Compound	Gram-Positive Bacteria		Gram-Negative Bacteria	
	*Staphylococcus aureus* ATCC25923	*Enterococcus faecalis* ATCC29212	*Pseudomonas aeruginosa* ATCC27853	*Escherichia coli* ATCC25922
**4a**	+	-	+	+
**4b**	+	+	+	+
**4c**	-	-	-	-
**4d**	-	-	-	-
**4e**	+	-	+	+
**4f**	+	-	+	+
**5a**	+	-	+	+
**5b**	+	+	+	+
**5c**	-	-	-	-
**5d**	-	-	-	-
**5e**	-	-	-	-
Metronidazole	+	+	+	+
Nitrofurantoin	+	+	+	+

The legend: the concentration of the compounds is 0.06 g/2 mL DMSO. (+) inhibited bacterial growth; (-) did not inhibit bacterial growth.

**Table 3:** Minimum inhibitory concentration (μg/mL) of the synthesized compounds of the two series **4a**–**f** and **5a**–**e**.

Compound	Gram-Positive Bacteria		Gram-Negative Bacteria	
	*Staphylococcus aureus* ATCC25923	*Enterococcus faecalis* ATCC29212	*Pseudomonas aeruginosa* ATCC27853	*Escherichia coli* ATCC25922
**4a**	230 ± 0.13	930 ± 0.25	930 ± 0.27	930 ± 0.14
**4b**	460 ± 0.02	460 ± 0.02	460 ± 0.01	460 ± 0.05
**4c**	930 ± 0.04	930 ± 0.03	930 ± 1.08	930 ± 1.05
**4d**	930 ± 0.55	930 ± 0.53	930 ± 0.02	930 ± 0.10
**4e**	460 ± 0.03	930 ± 0.02	930 ± 0.53	460 ± 0.52
**4f**	460 ± 0.01	930 ± 0.04	460 ± 0.03	460 ± 0.02
**5a**	460 ± 0.03	930 ± 0.06	460 ± 0.04	460 ± 0.06
**5b**	460 ± 0.01	460 ± 0.54	460 ± 0.58	460 ± 0.27
**5c**	3750 ± 0.04	3750 ± 0.08	3750 ± 0.51	3750 ± 0.54
**5d**	1870 ± 0.07	1870 ± 0.05	1870 ± 0.04	1870 ± 0.04
**5e**	930 ± 0.04	1870 ± 0.09	1870 ± 0.04	1870 ± 0.08
Metronidazole	1950 ± 0.05	970 ± 0.17	1562 ± 0.03	1950 ± 0.05
Nitrofurantoin	600 ± 0.04	600 ± 0.12	3571 ± 0.08	600 ± 0.02

**Table 4:** Minimum biofilm inhibition concentration (MBIC) for the synthesized compounds of the two series **4a**–**f** and **5a**–**e**.

Compound	Gram-Positive Bacteria		Gram-Negative Bacteria	
	*Staphylococcus aureus* ATCC25923	*Enterococcus faecalis* ATCC29212	*Pseudomonas aeruginosa* ATCC27853	*Escherichia coli* ATCC25922
**4a**	230 ± 0.11	930 ± 0.27	930 ± 0.28	930 ± 0.05
**4b**	460 ± 0.01	930 ± 0.15	460 ± 0.02	930 ± 0.04
**4c**	930 ± 0.06	930 ± 1.09	930 ± 0.52	930 ± 0.11
**4d**	930 ± 0.03	930 ± 0.53	460 ± 0.05	930 ± 0.18
**4e**	460 ± 0.04	930 ± 0.04	930 ± 0.02	930 ± 0.09
**4f**	460 ± 0.02	930 ± 0.03	460 ± 0.01	460 ± 0.02
**5a**	230 ± 0.05	930 ± 0.14	460 ± 0.12	460 ± 0.12
**5b**	460 ± 0.01	930 ± 0.09	460 ± 0.51	460 ± 0.03
**5c**	1870 ± 0.15	1870 ± 0.06	1870 ± 0.53	3750 ± 0.48
**5d**	930 ± 0.15	1870 ± 0.15	930 ± 0.15	1870 ± 0.15
**5e**	930 ± 0.15	930 ± 0.15	930 ± 0.15	930 ± 0.15
Metronidazole	1950 ± 0.09	970 ± 0.21	1562 ± 0.11	1950 ± 0.04
Nitrofurantoin	2230 ± 0.13	600 ± 0.17	550 ± 0.23	600 ± 0.03

## Data Availability

The data presented in this study are available on request from the corresponding author.

## Supplementary Materials

The following supporting information can be downloaded at: https://www.mdpi.com/article/10.3390/antibiotics13121119/s1. Online supplementary information includes detailed spectra of ^1^H-NMR and ^13^C-NMR for the compounds **4a**–**f** and **5a**–**e** and XRD for **4a** and **5d** compounds.
