# Supplementary material for: Synthesis, Spectroscopic Characterization, Structural Analysis, and Evaluation of Anti-Tumor, Antimicrobial, and Antibiofilm Activities of Halogenoaminopyrazoles Derivatives
Source: Antibiotics (Basel). 2024 Nov 22;13(12):1119. doi: 10.3390/antibiotics13121119 (PMC11672400; doi:10.3390/antibiotics13121119)
Supplement: Supplementary file 1 [file antibiotics-13-01119-s001.zip › antibiotics-3269316-supplementary.pdf]

4a ¹H-NMR

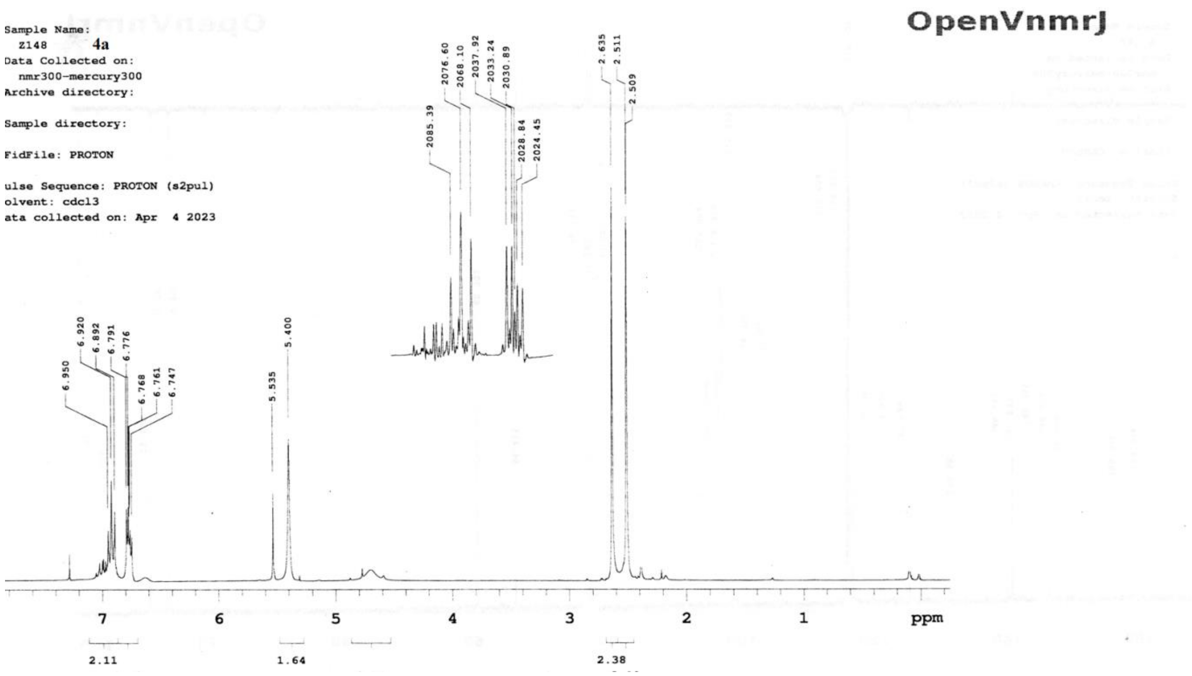

4a ¹³C-NMR

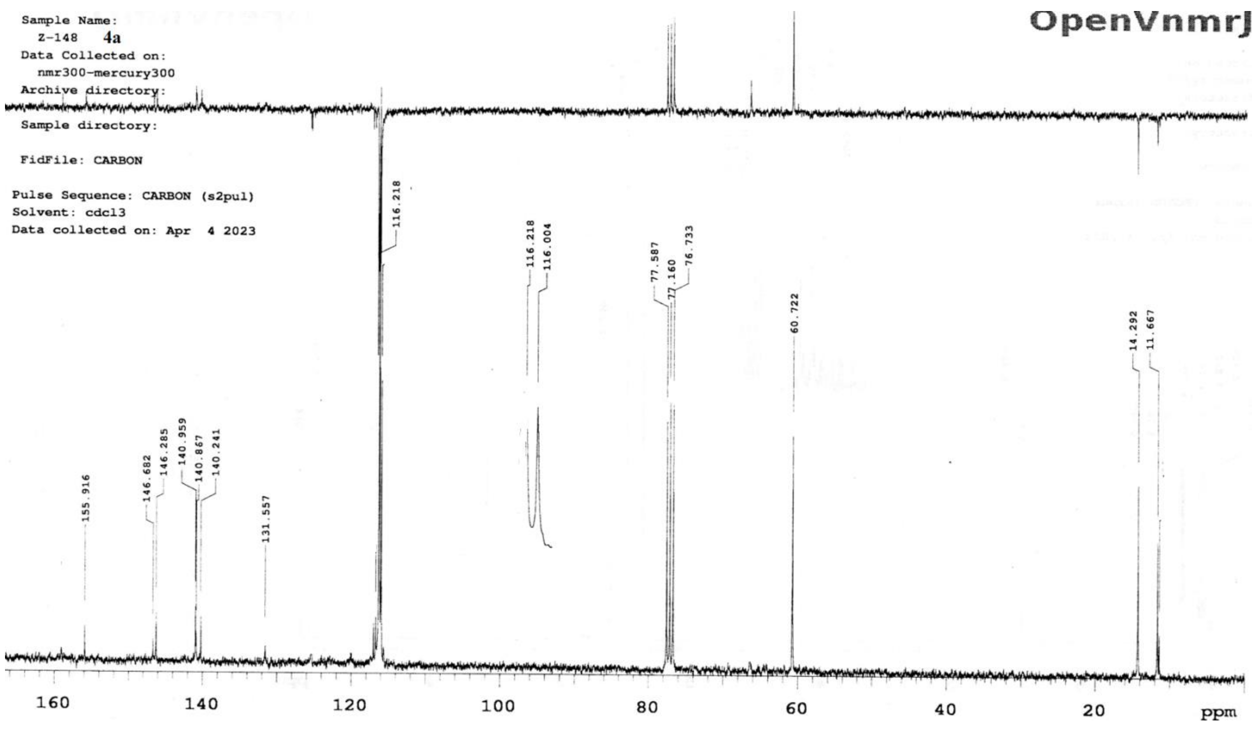

4b ¹H-NMR

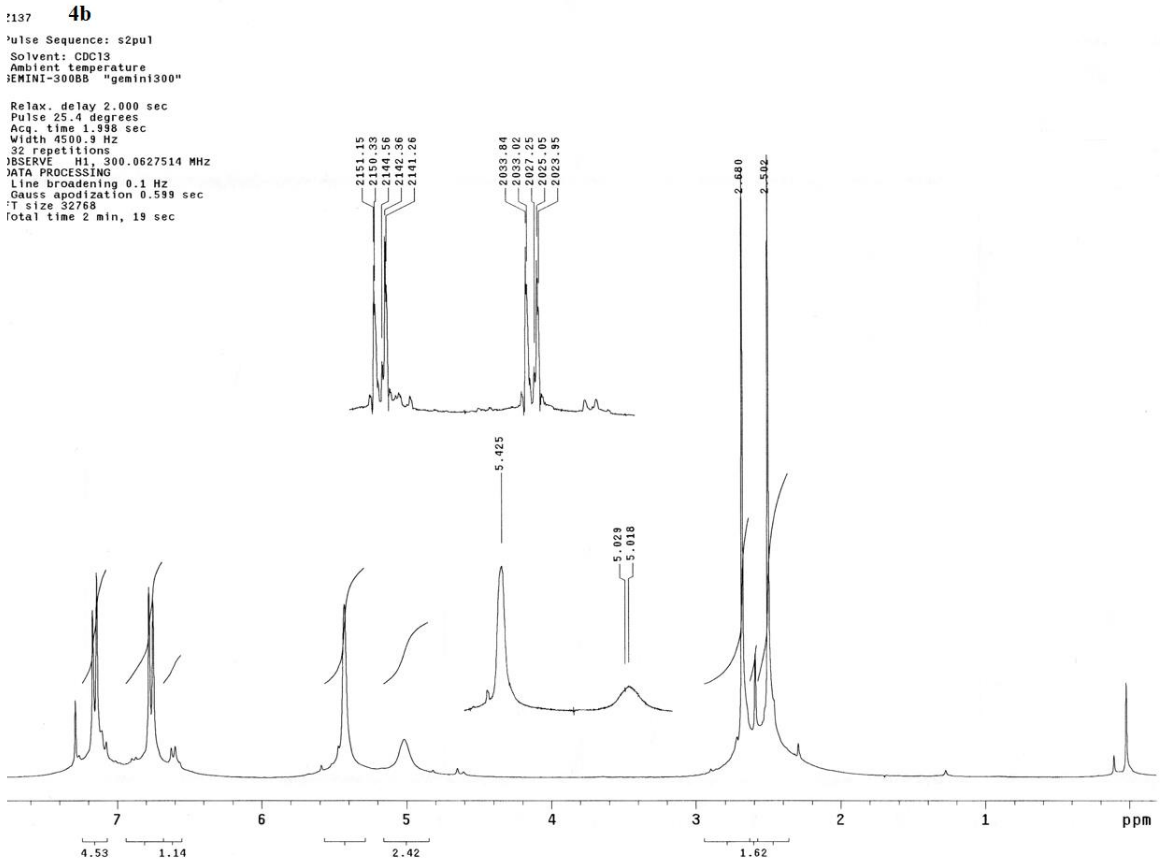

2137 4b
Pulse Sequence: s2pul
Solvent: CDCl3
Ambient temperature
GEMINI-300BB "gemini300"
Relax. delay 2.000 sec
Pulse 25.4 degrees
Acq. time 1.998 sec
Width 4500.9 Hz
32 repetitions
OBSERVE H1, 300.0627514 MHz
DATA PROCESSING
Line broadening 0.1 Hz
Gauss apodization 0.599 sec
FT size 32768
Total time 2 min, 19 sec
2151.15
2150.33
2144.56
2142.36
2141.26
2033.84
2033.02
2027.25
2025.05
2023.95
2.680
2.502
5.425
5.029
5.018
7
6
5
4
3
2
1
ppm
4.53
1.14
2.42
1.62

4b ^{13}C-NMR

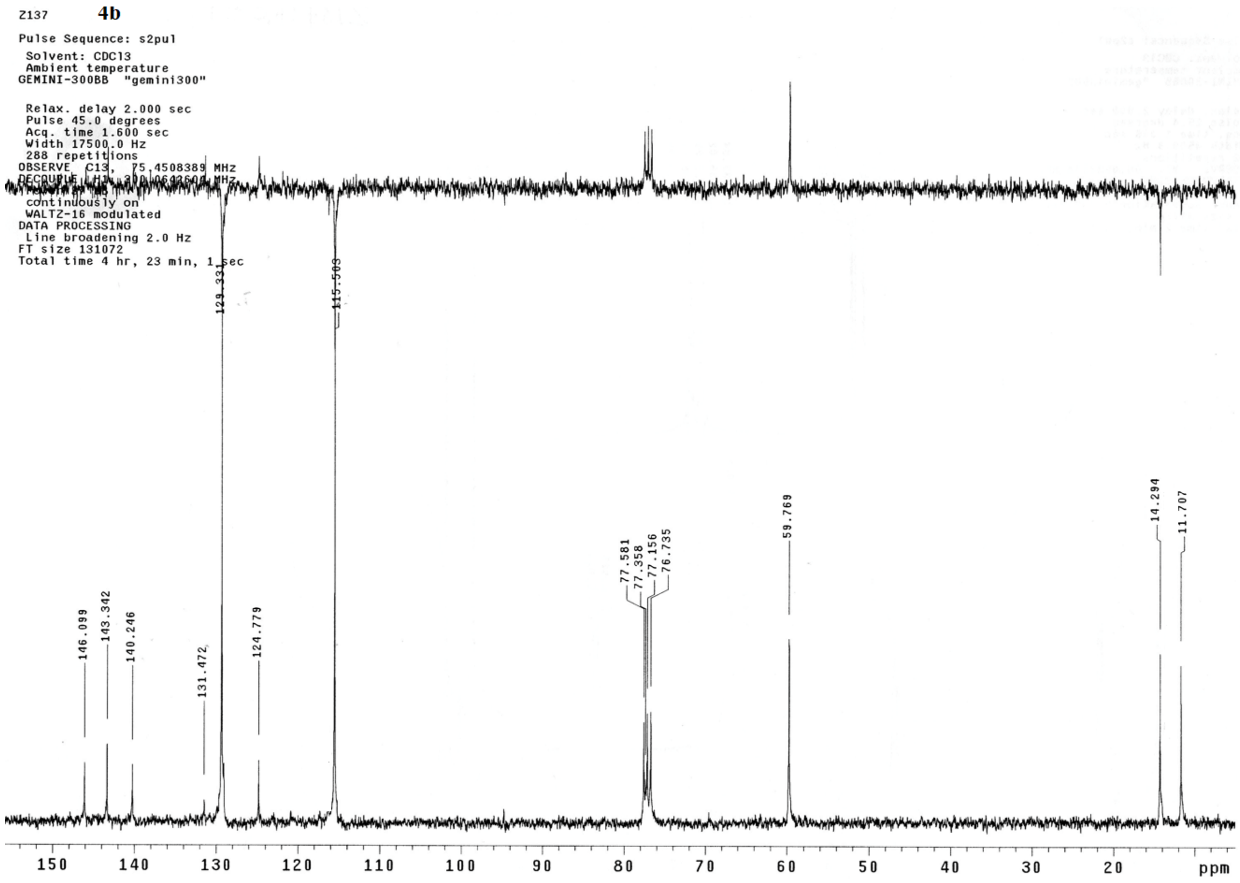
Z137 4b
Pulse Sequence: s2pul
Solvent: CDCl3
Ambient temperature
GEMINI-300BB "gemini300"
Relax. delay 2.000 sec
Pulse 45.0 degrees
Acq. time 1.600 sec
Width 17500.0 Hz
288 repetitions
OBSERVE C13, 75.4508389 MHz
continuously on
WALTZ-16 modulated
DATA PROCESSING
Line broadening 2.0 Hz
FT size 131072
Total time 4 hr, 23 min, 1 sec
146.099
143.342
140.246
131.472
129.331
124.779
115.503
77.581
77.358
77.156
76.735
59.769
14.294
11.707
150 140 130 120 110 100 90 80 70 60 50 40 30 20 ppm

4c ^{1}H-NMR

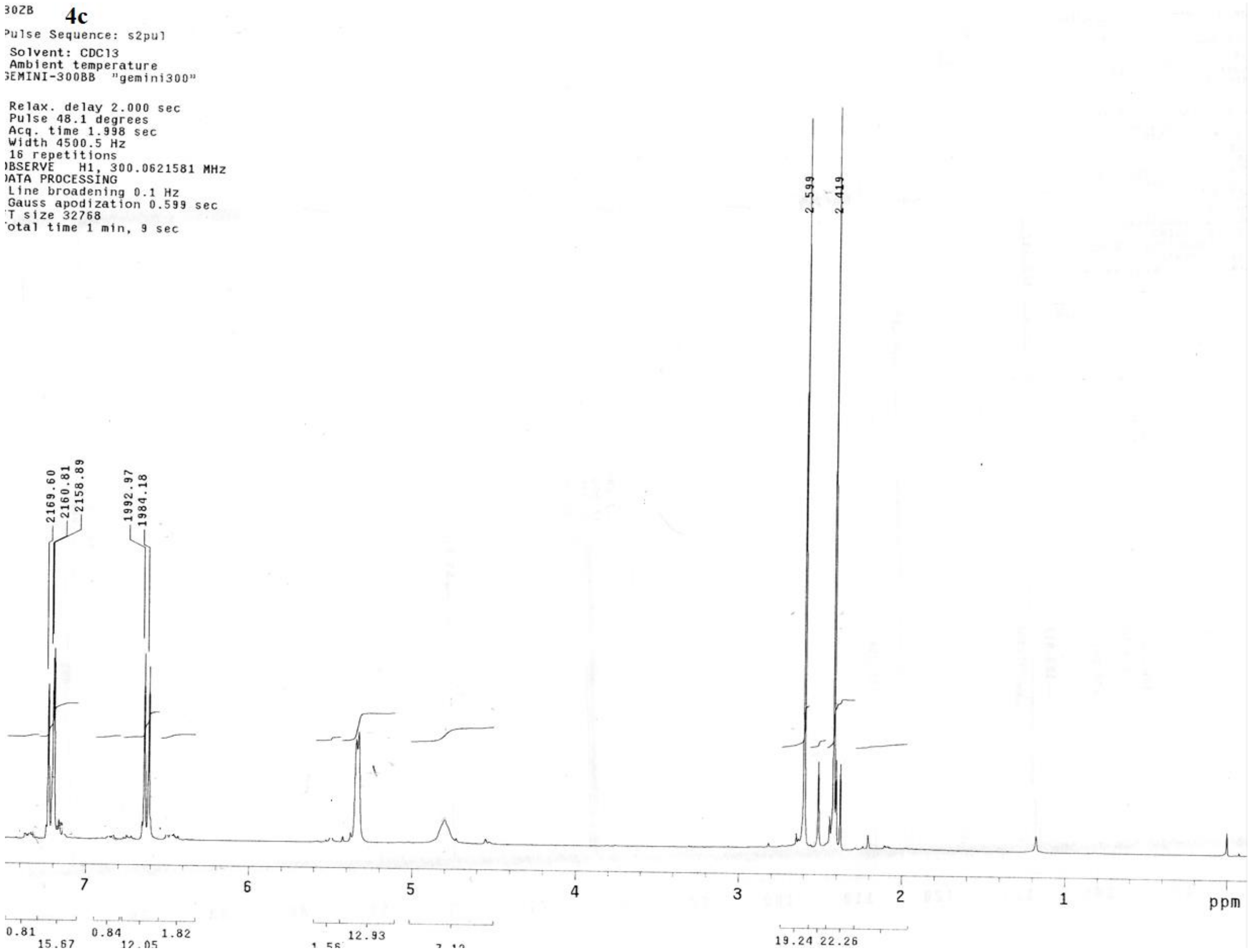

30ZB
4c
Pulse Sequence: s2pul
Solvent: CDCl3
Ambient temperature
GEMINI-300BB "gemini300"
Relax. delay 2.000 sec
Pulse 48.1 degrees
Acq. time 1.998 sec
Width 4500.5 Hz
16 repetitions
OBSERVE H1, 300.0621581 MHz
DATA PROCESSING
Line broadening 0.1 Hz
Gauss apodization 0.599 sec
FT size 32768
Total time 1 min, 9 sec
2169.60
2160.81
2158.89
1992.97
1984.18
2.599
2.419
7
6
5
4
3
2
1
ppm
0.81
15.67
0.84
12.05
1.82
12.93
19.24
22.26

4c ^{13}C-NMR

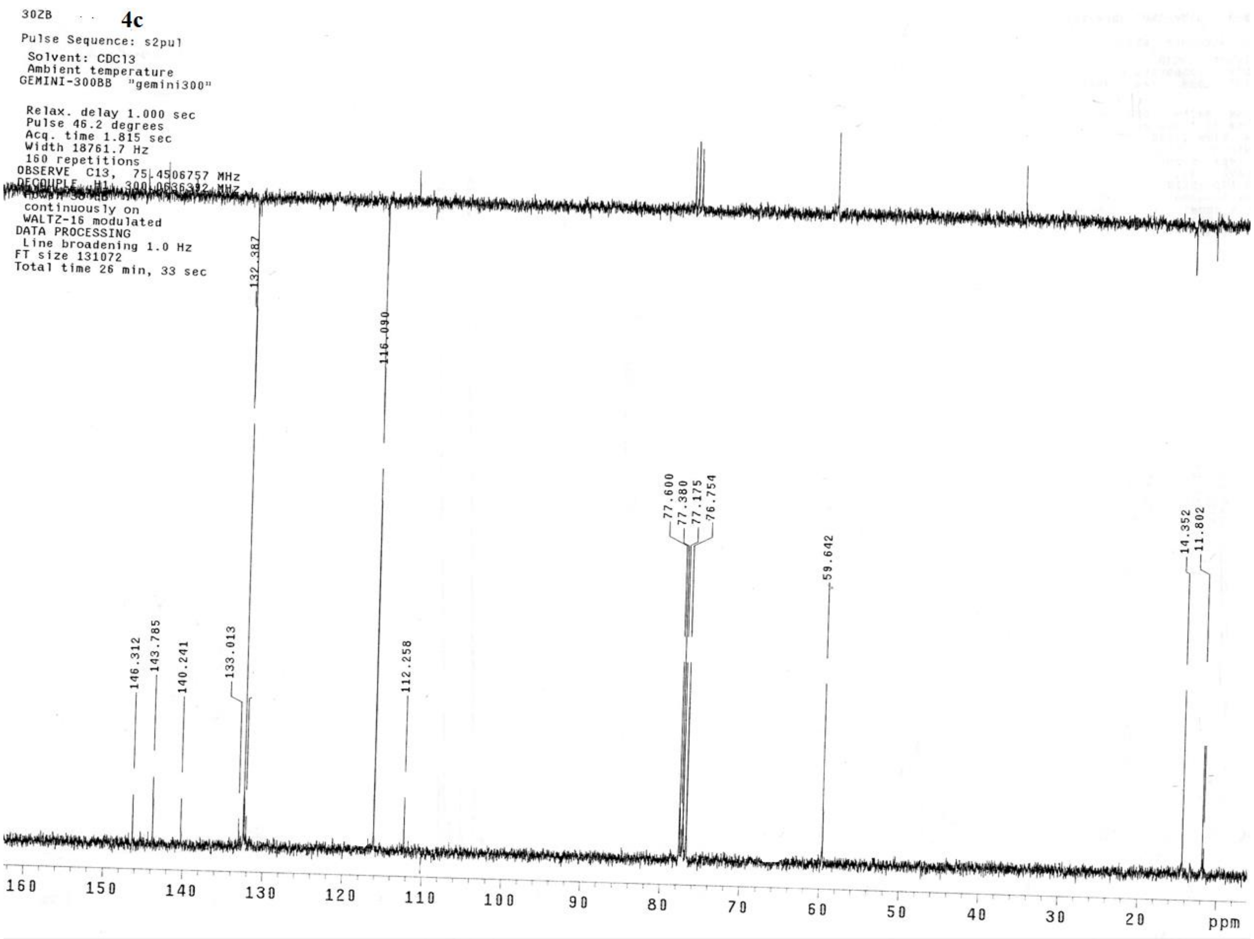

302B 4c
Pulse Sequence: s2pul
Solvent: CDCl3
Ambient temperature
GEMINI-300BB "gemini300"
Relax. delay 1.000 sec
Pulse 46.2 degrees
Acq. time 1.815 sec
Width 18761.7 Hz
160 repetitions
OBSERVE C13, 75.4506757 MHz
DECOUPLE H1, 300.0636392 MHz
continuously on
WALTZ-16 modulated
DATA PROCESSING
Line broadening 1.0 Hz
FT size 131072
Total time 26 min, 33 sec
132.387
116.090
146.312
143.785
140.241
133.013
112.258
77.600
77.380
77.175
76.754
59.642
14.352
11.802
ppm

4d ¹H-NMR

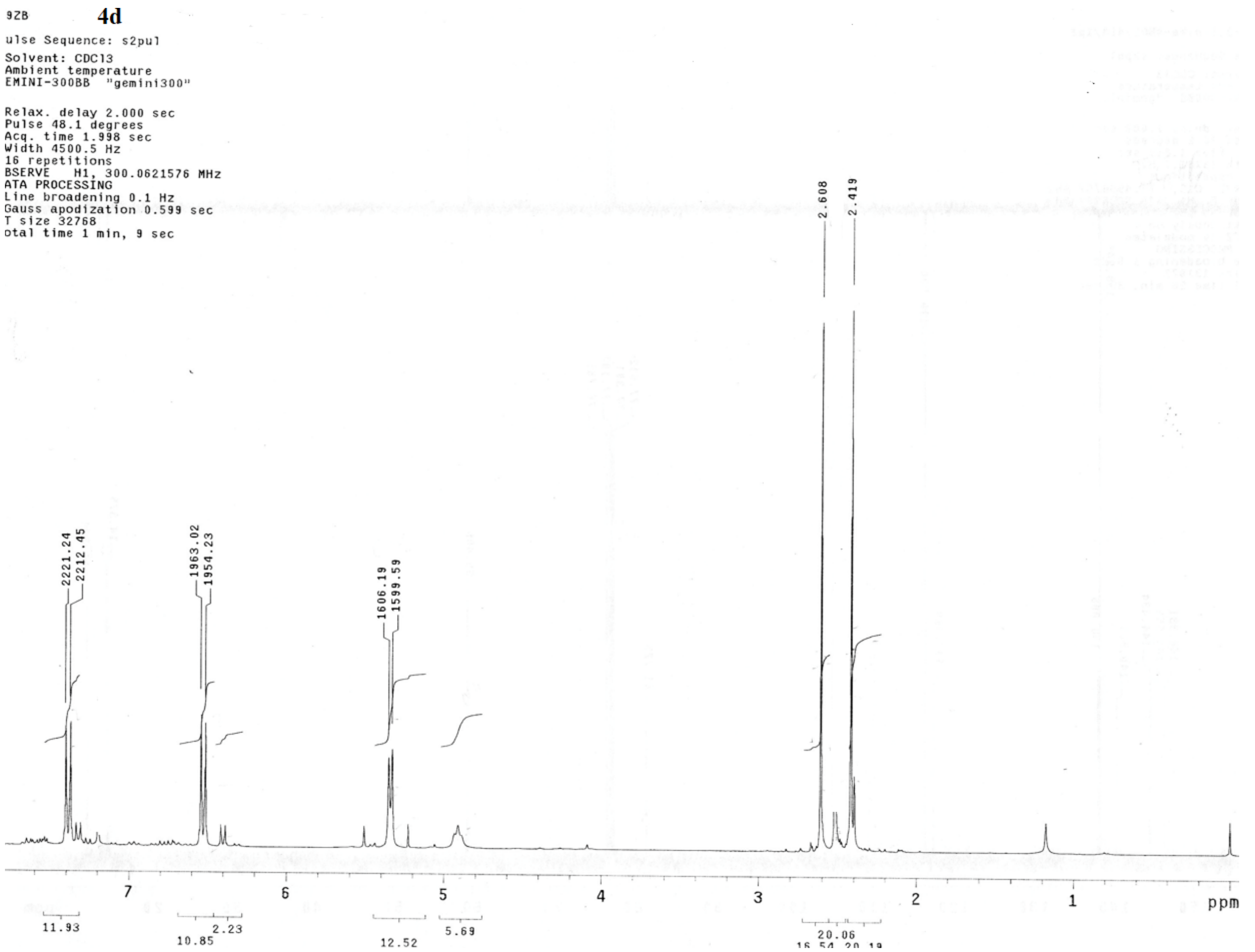
9ZB 4d
ulse Sequence: s2pul
Solvent: CDCl3
Ambient temperature
EMINI-300BB "gemini300"
Relax. delay 2.000 sec
Pulse 48.1 degrees
Acq. time 1.998 sec
Width 4500.5 Hz
16 repetitions
BSERVE H1, 300.0621576 MHz
ATA PROCESSING
Line broadening 0.1 Hz
Gauss apodization 0.599 sec
T size 32768
otal time 1 min, 9 sec
2.608
2.419
2221.24
2212.45
1963.02
1954.23
1606.19
1599.59
7
6
5
4
3
2
1
ppm
11.93
10.85
2.23
12.52
5.69
20.06

4d ^{13}C-NMR

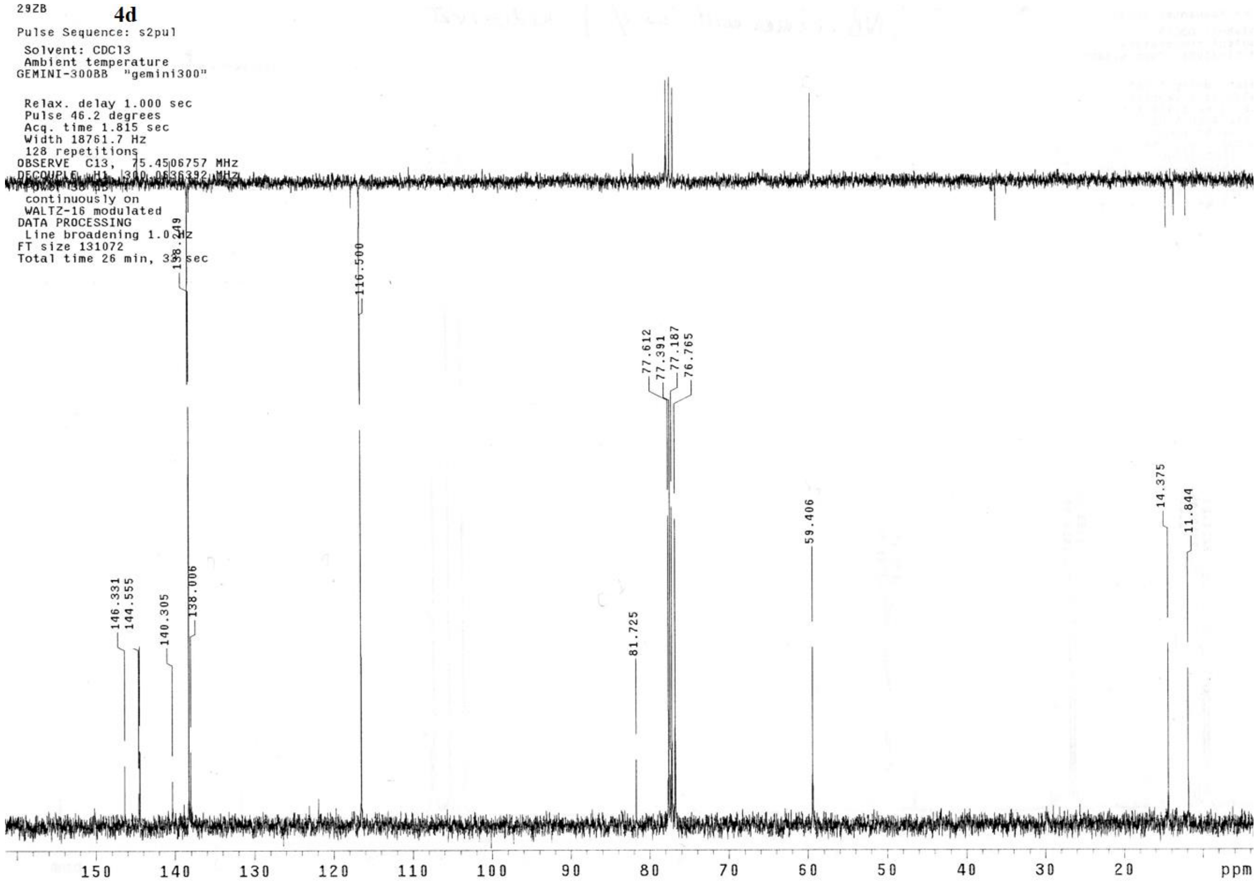
29ZB
4d
Pulse Sequence: s2pul
Solvent: CDCl3
Ambient temperature
GEMINI-300BB "gemini300"
Relax. delay 1.000 sec
Pulse 46.2 degrees
Acq. time 1.815 sec
Width 18761.7 Hz
128 repetitions
OBSERVE C13, 75.4506757 MHz
continuously on
WALTZ-16 modulated
DATA PROCESSING
FT size 131072
146.331
144.555
140.305
138.006
116.500
81.725
77.612
77.391
77.187
76.765
59.406
14.375
11.844
150
140
130
120
110
100
90
80
70
60
50
40
30
20
ppm

4e ^{1}H-NMR

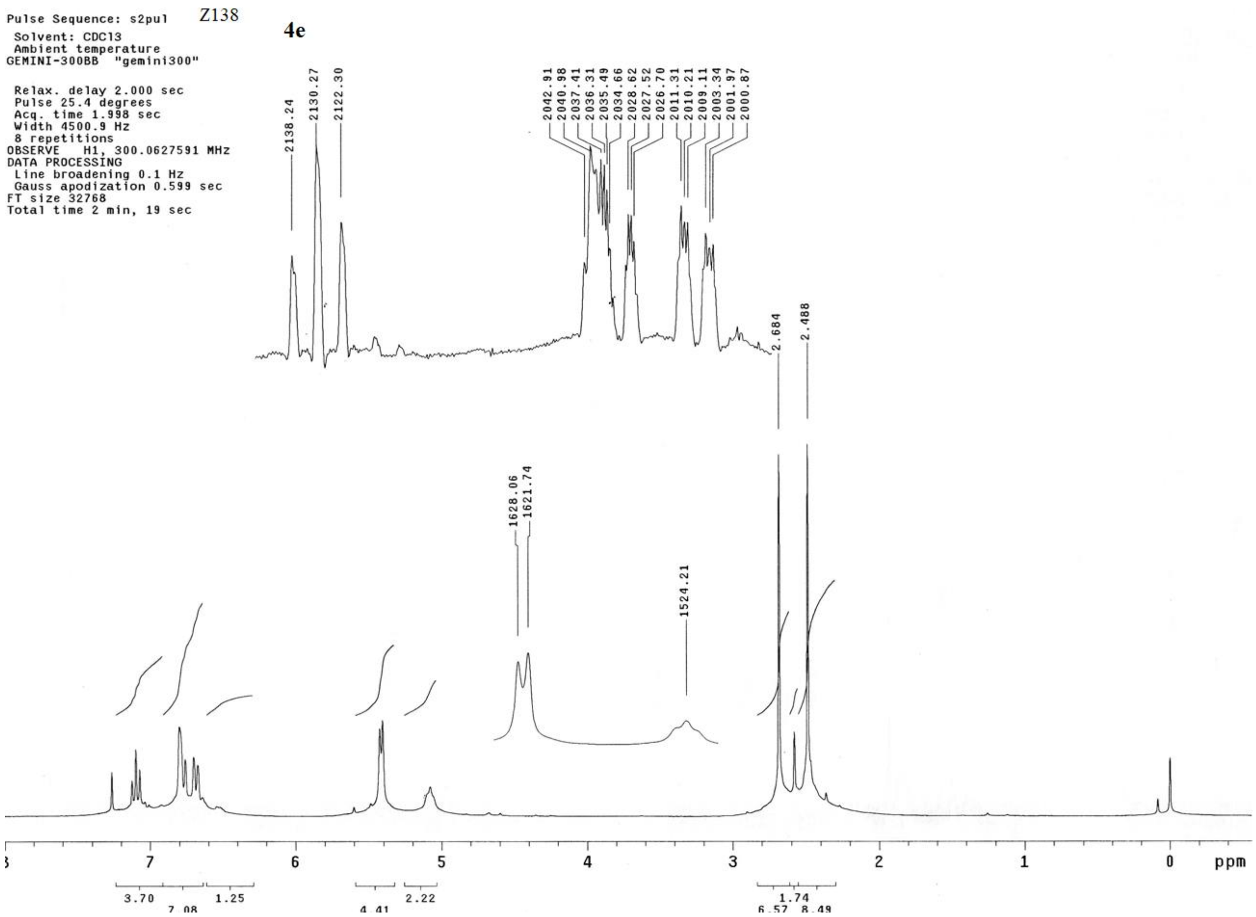

Pulse Sequence: s2pul
Z138
Solvent: CDCl3
Ambient temperature
GEMINI-300BB "gemini300"
Relax. delay 2.000 sec
Pulse 25.4 degrees
Acq. time 1.998 sec
Width 4500.9 Hz
8 repetitions
OBSERVE H1, 300.0627591 MHz
DATA PROCESSING
Line broadening 0.1 Hz
Gauss apodization 0.599 sec
FT size 32768
Total time 2 min, 19 sec
4e
2138.24
2130.27
2122.30
2042.91
2040.98
2037.41
2036.31
2035.49
2034.66
2028.62
2027.52
2026.70
2011.31
2010.21
2009.11
2003.34
2001.97
2000.87
2.684
2.488
1628.06
1621.74
1524.21
ppm
3.70
7.08
1.25
4.41
2.22
1.74
6.57
8.49

4e ^{13}C-NMR

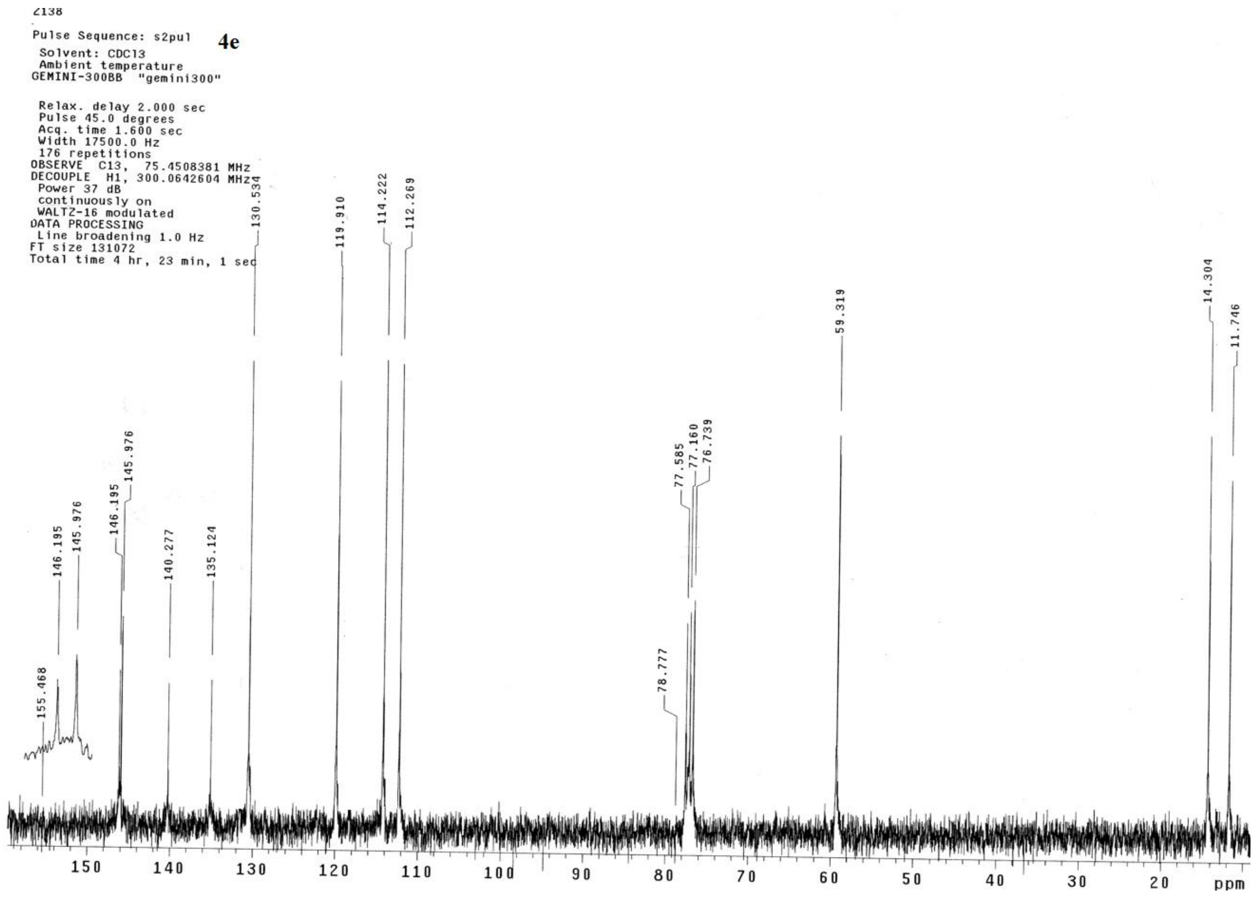

2138
Pulse Sequence: s2pul
4e
Solvent: CDCl3
Ambient temperature
GEMINI-300BB "gemini300"
Relax. delay 2.000 sec
Pulse 45.0 degrees
Acq. time 1.600 sec
Width 17500.0 Hz
176 repetitions
OBSERVE C13, 75.4508381 MHz
DECOUPLE H1, 300.0642604 MHz
Power 37 dB
continuously on
WALTZ-16 modulated
DATA PROCESSING
Line broadening 1.0 Hz
FT size 131072
Total time 4 hr, 23 min, 1 sec
155.468
146.195
145.976
146.195
145.976
140.277
135.124
130.534
119.910
114.222
112.269
78.777
77.585
77.160
76.739
59.319
14.304
11.746
150
140
130
120
110
100
90
80
70
60
50
40
30
20
ppm

4f ¹H-NMR

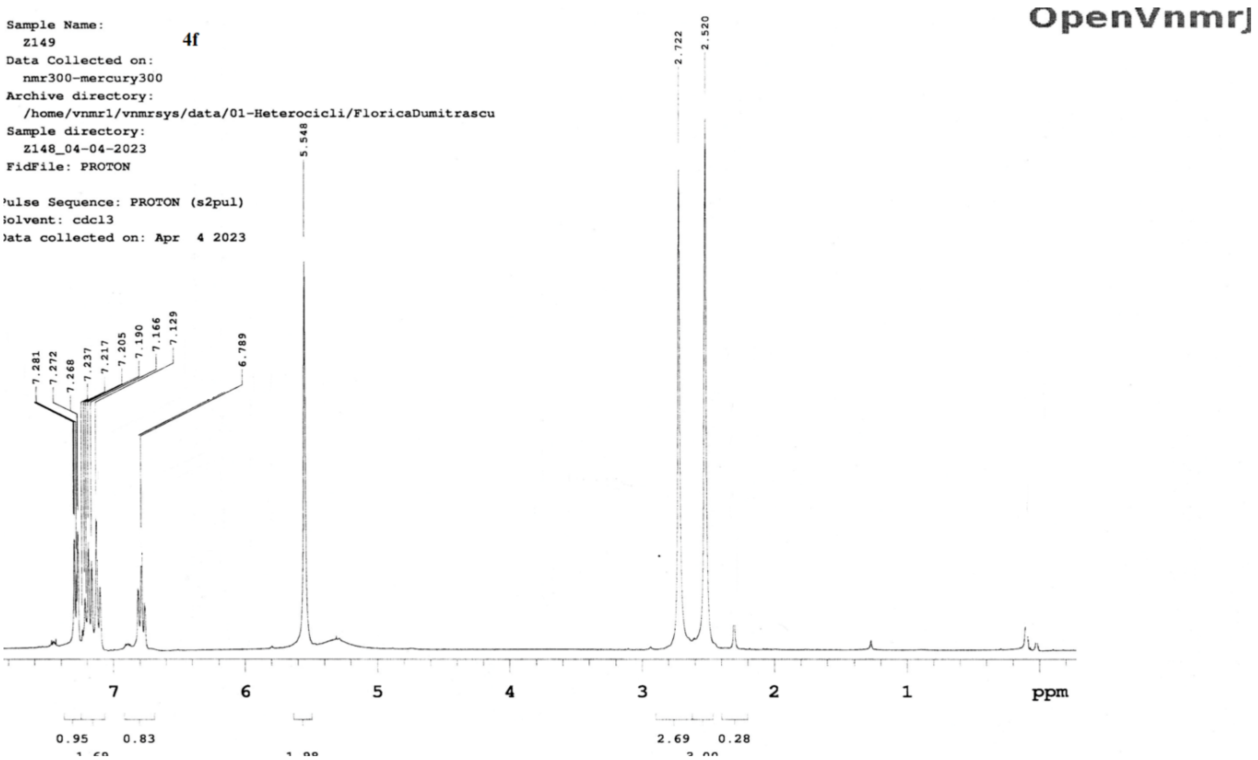
Sample Name:
Z149
4f
Data Collected on:
nmr300-mercury300
Archive directory:
/home/vnmr1/vnmrsys/data/01-Heterocicli/FloricaDumitrascu
Sample directory:
Z148_04-04-2023
FidFile: PROTON
Pulse Sequence: PROTON (s2pul)
Solvent: cdcl3
Data collected on: Apr 4 2023
OpenVnmrJ
7.281
7.272
7.268
7.237
7.217
7.205
7.190
7.166
7.129
6.789
5.548
2.722
2.520
7
6
5
4
3
2
1
ppm
0.95
0.83
2.69
0.28

4f ^{13}C-NMR

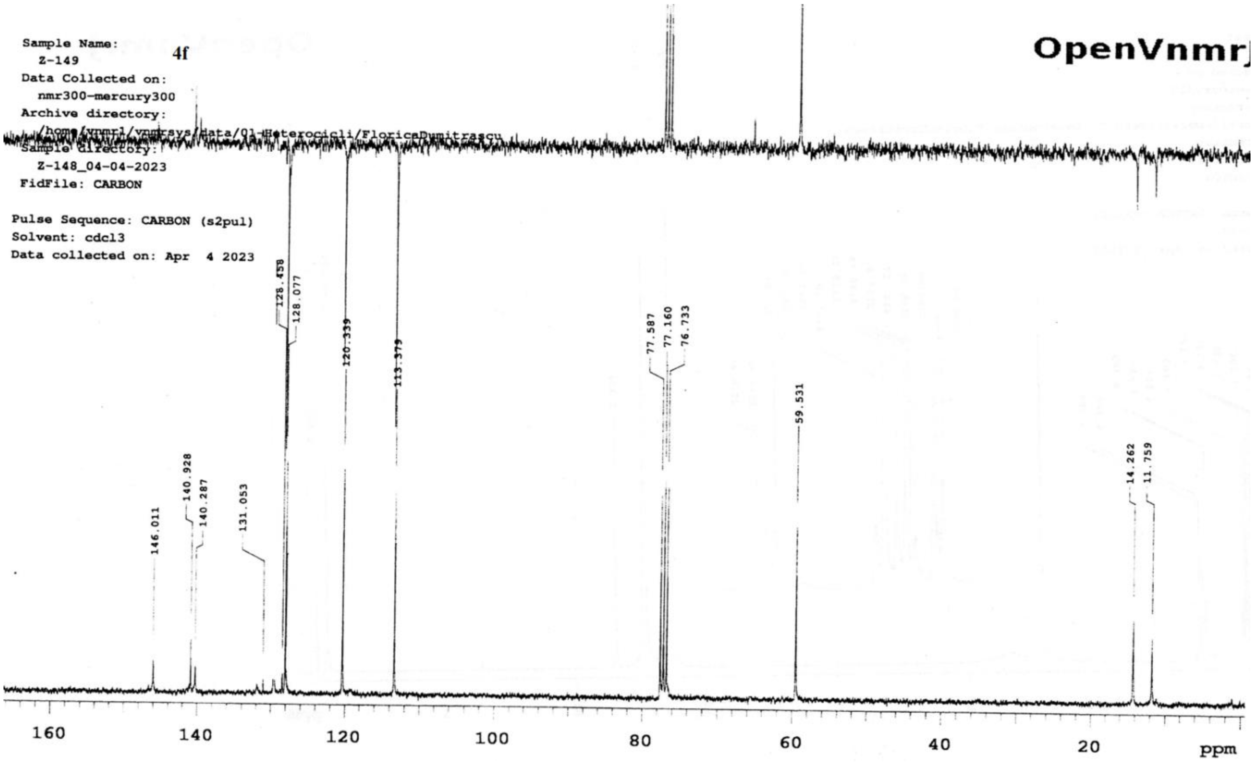

Sample Name:
Z-149
4f
Data Collected on:
nmr300-mercury300
Archive directory:
Sample directory:
Z-148_04-04-2023
FidFile: CARBON
Pulse Sequence: CARBON (s2pul)
Solvent: cdcl3
Data collected on: Apr 4 2023
OpenVnmrJ
146.011
140.928
140.287
131.053
128.458
128.077
120.339
113.379
77.587
77.160
76.733
59.531
14.262
11.759
160
140
120
100
80
60
40
20
ppm

5a ¹H-NMR

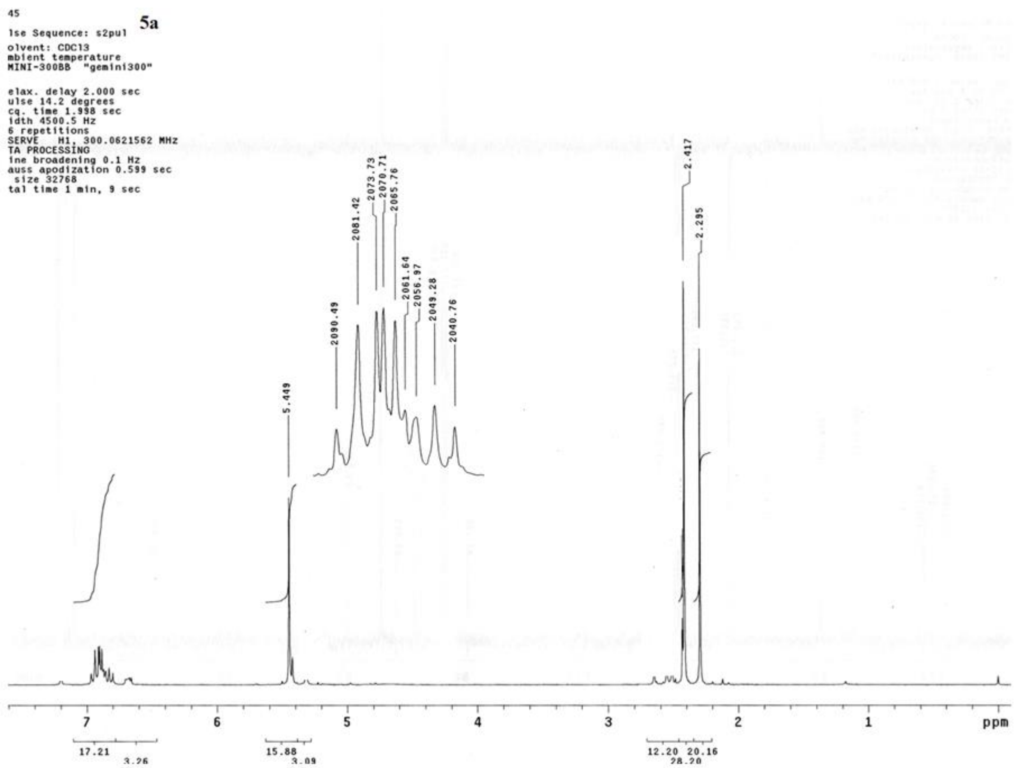

45
lse Sequence: s2pul
5a
olvent: CDCl3
mbient temperature
MINI-300BB "gemini300"
elax. delay 2.000 sec
ulse 14.2 degrees
cq. time 1.998 sec
idth 4500.5 Hz
6 repetitions
SERVE H1, 300.0621562 MHz
TA PROCESSING
ine broadening 0.1 Hz
auss apodization 0.599 sec
size 32768
tal time 1 min, 9 sec
5.449
2090.49
2081.42
2073.73
2070.71
2065.76
2061.64
2056.97
2049.28
2040.76
2.417
2.295
7
6
5
4
3
2
1
ppm
17.21
3.26
15.88
3.09
12.20
20.16
28.20

5a ^{13}C-NMR

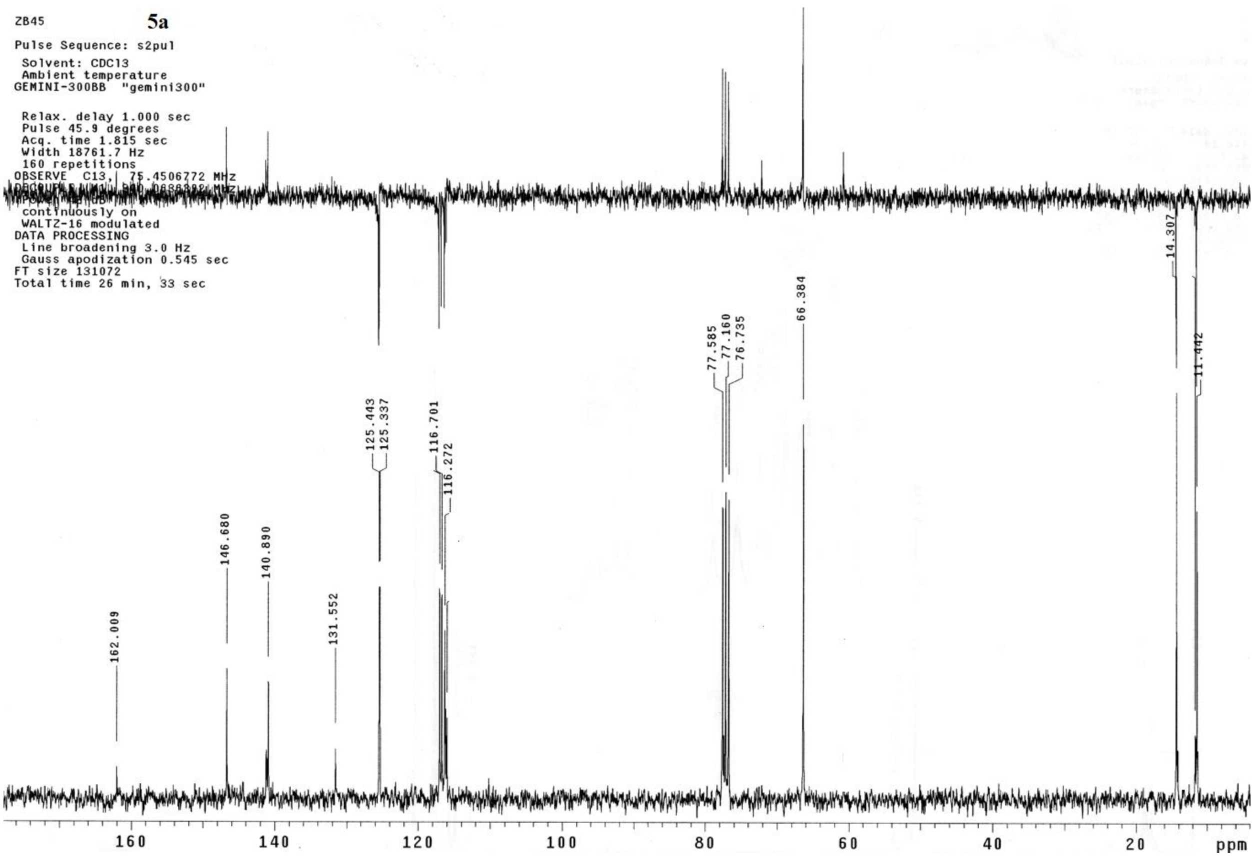
ZB45
5a
Pulse Sequence: s2pul
Solvent: CDC13
Ambient temperature
GEMINI-300BB "gemini300"
Relax. delay 1.000 sec
Pulse 45.9 degrees
Acq. time 1.815 sec
Width 18761.7 Hz
160 repetitions
OBSERVE C13, 75.4506772 MHz
continuously on
WALTZ-16 modulated
DATA PROCESSING
Line broadening 3.0 Hz
Gauss apodization 0.545 sec
FT size 131072
Total time 26 min, 33 sec
162.009
146.680
140.890
131.552
125.443
125.337
116.701
116.272
77.585
77.160
76.735
66.384
14.307
11.442
160
140
120
100
80
60
40
20
ppm

5b ¹H-NMR

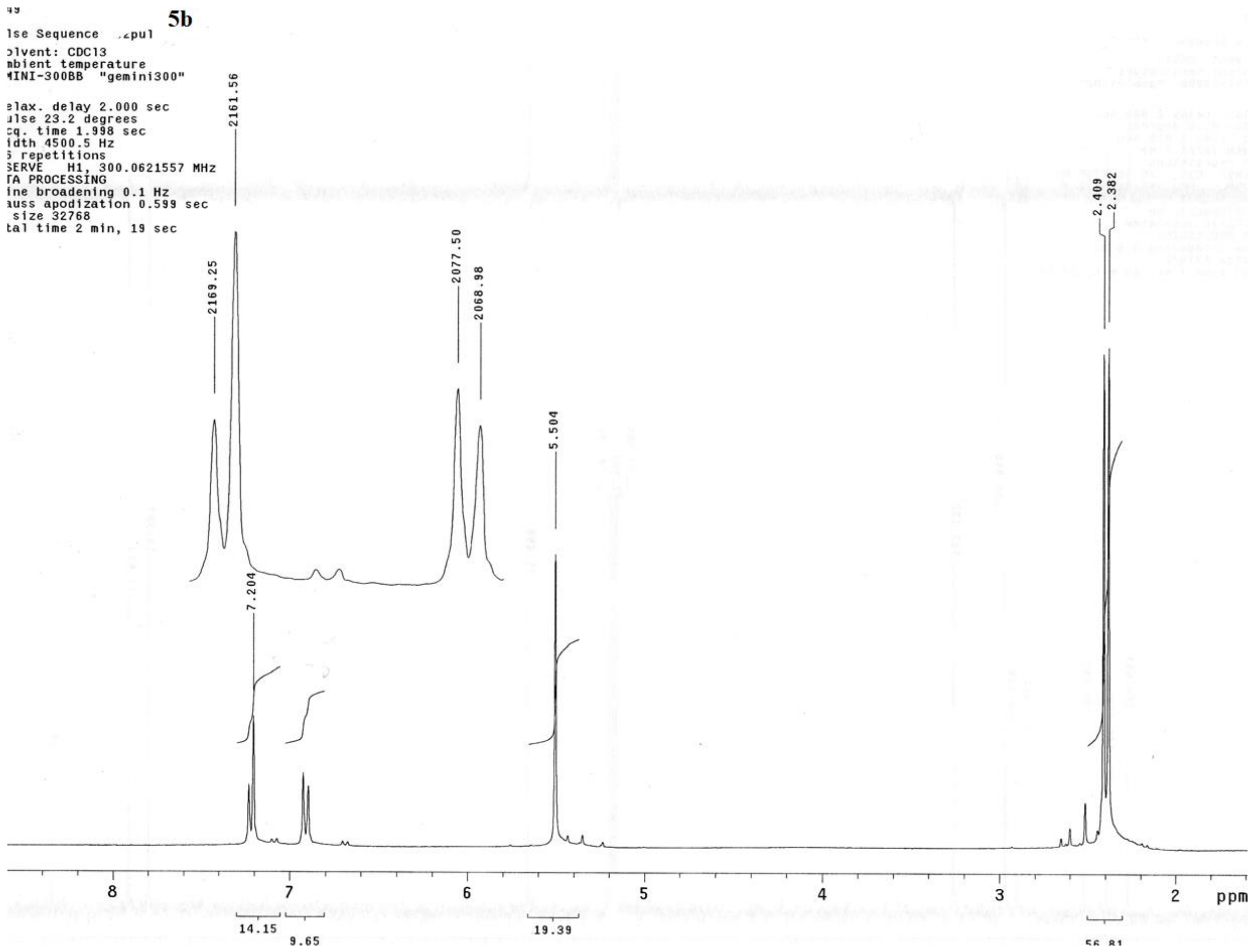

5b
lse Sequence .2pul
olvent: CDCl3
mbient temperature
MINI-300BB "gemini300"
elax. delay 2.000 sec
ulse 23.2 degrees
cq. time 1.998 sec
idth 4500.5 Hz
5 repetitions
SERVE H1, 300.0621557 MHz
TA PROCESSING
ine broadening 0.1 Hz
auss apodization 0.599 sec
size 32768
tal time 2 min, 19 sec
2161.56
2169.25
2077.50
2068.98
5.504
7.204
2.409
2.382
8
7
6
5
4
3
2
ppm
14.15
9.65
19.39

5b ^{13}C-NMR

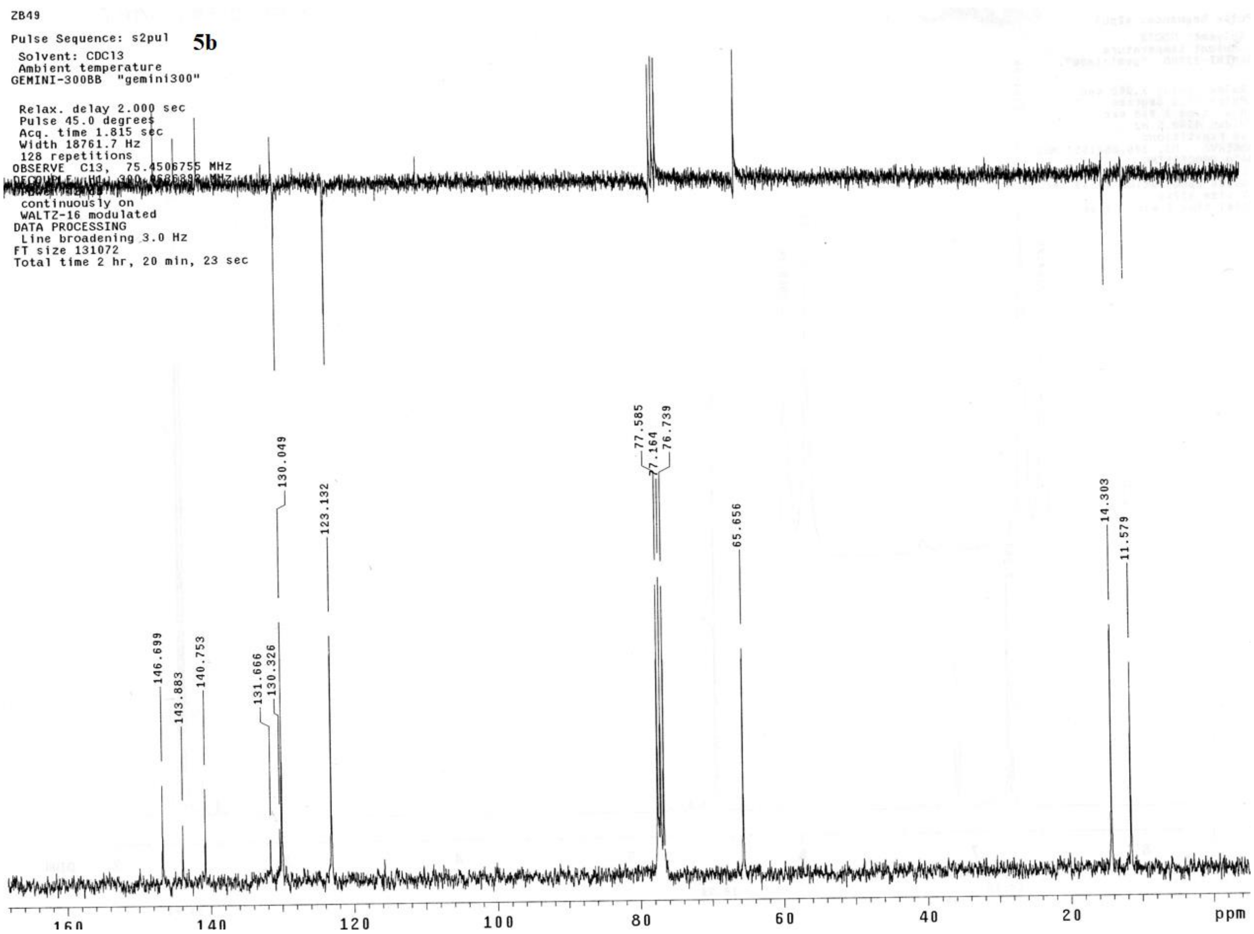
ZB49
Pulse Sequence: s2pul
5b
Solvent: CDCl3
Ambient temperature
GEMINI-300BB "gemini300"
Relax. delay 2.000 sec
Pulse 45.0 degrees
Acq. time 1.815 sec
Width 18761.7 Hz
128 repetitions
OBSERVE C13, 75.4506755 MHz
continuously on
WALTZ-16 modulated
DATA PROCESSING
Line broadening 3.0 Hz
FT size 131072
Total time 2 hr, 20 min, 23 sec
146.699
143.883
140.753
131.666
130.326
130.049
123.132
77.585
77.164
76.739
65.656
14.303
11.579
160
140
120
100
80
60
40
20
ppm

5c ^{1}H-NMR

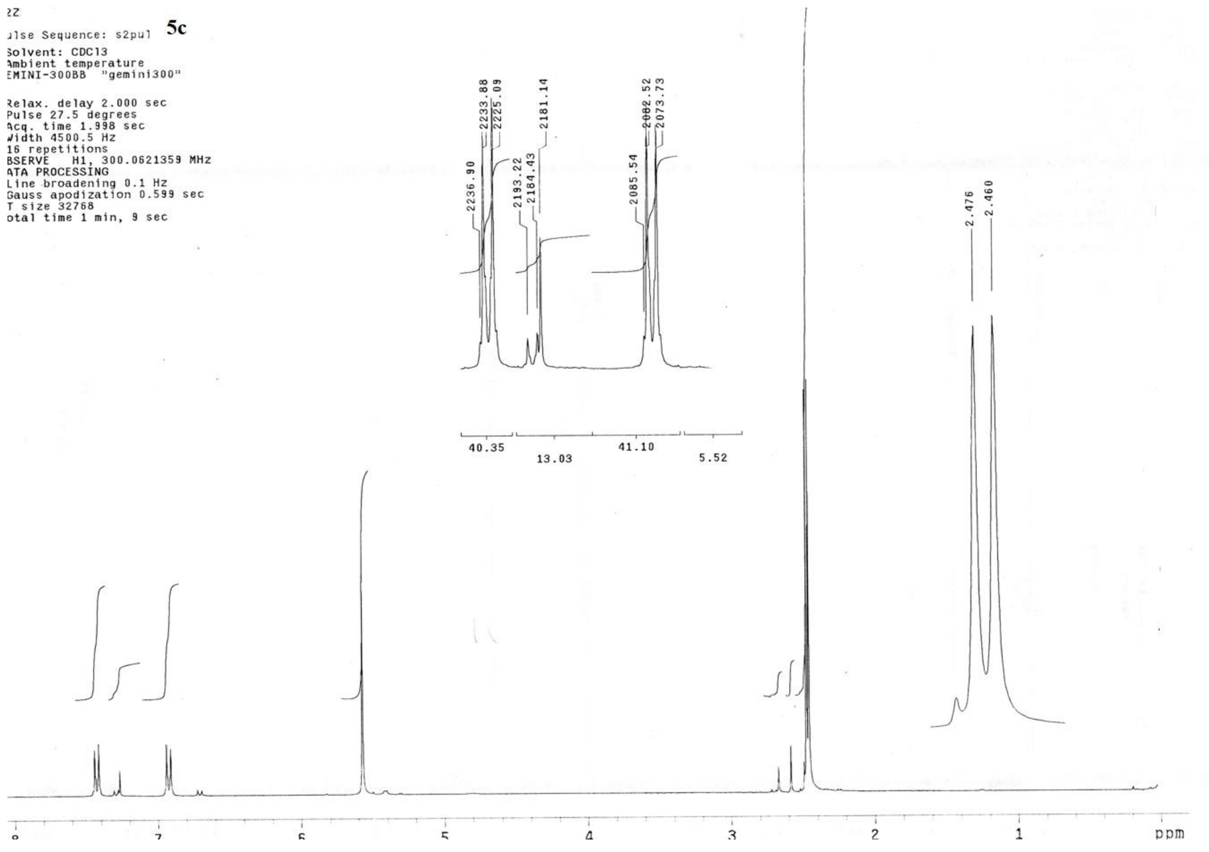

5c
ılse Sequence: s2pul
Solvent: CDCl3
Ambient temperature
EMINI-300BB "gemini300"
Relax. delay 2.000 sec
Pulse 27.5 degrees
Acq. time 1.998 sec
Width 4500.5 Hz
16 repetitions
BSERVE H1, 300.0621359 MHz
ATA PROCESSING
Line broadening 0.1 Hz
Gauss apodization 0.599 sec
T size 32768
otal time 1 min, 9 sec
2236.90
2233.88
2225.09
2193.22
2184.43
2181.14
2085.54
2082.52
2073.73
40.35
13.03
41.10
5.52
2.476
2.460
7
6
5
4
3
2
1
ppm

5c ^{13}C-NMR

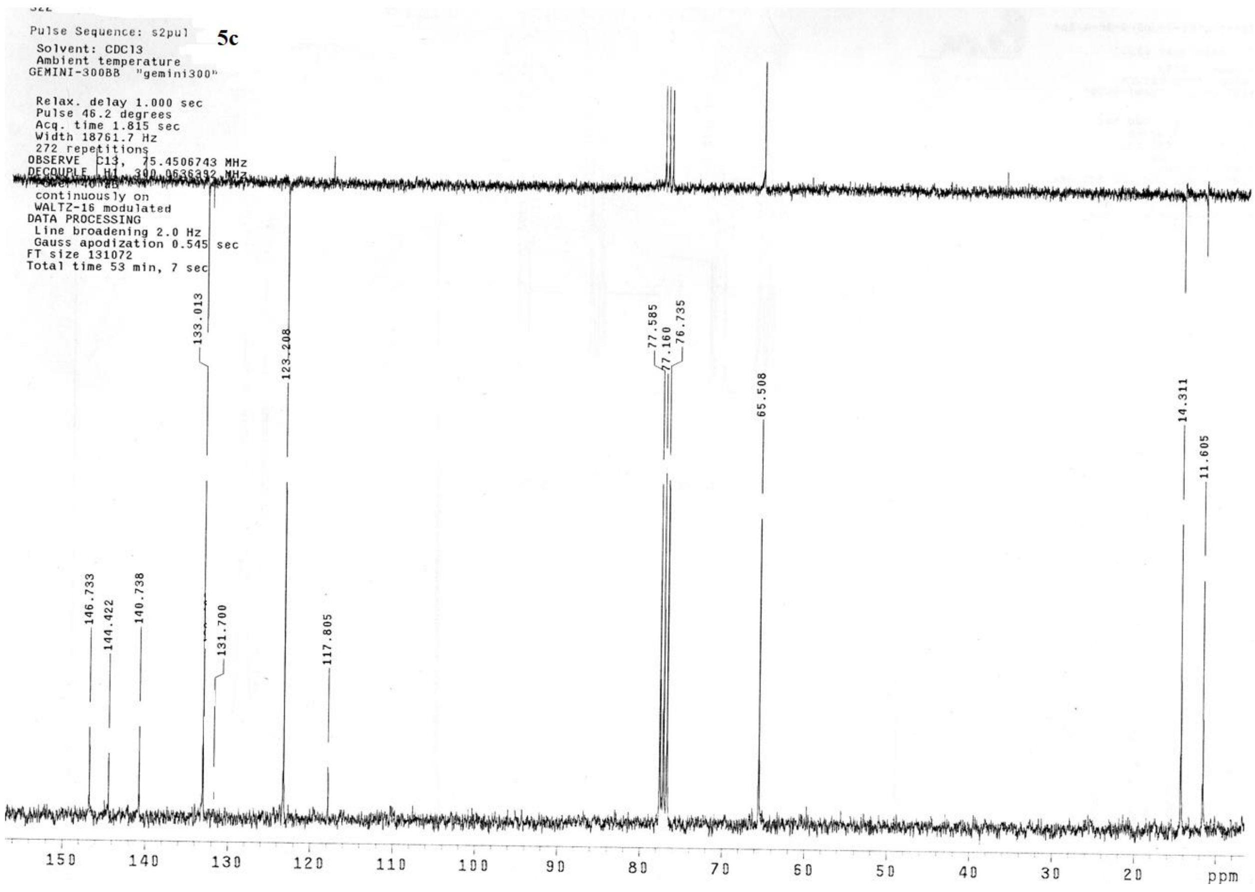
Pulse Sequence: s2pul
5c
Solvent: CDCl3
Ambient temperature
GEMINI-300BB "gemini300"
Relax. delay 1.000 sec
Pulse 46.2 degrees
Acq. time 1.815 sec
Width 18761.7 Hz
272 repetitions
OBSERVE C13, 75.4506743 MHz
DECOUPLE H1, 300.0636632 MHz
continuously on
WALTZ-16 modulated
DATA PROCESSING
Line broadening 2.0 Hz
Gauss apodization 0.545 sec
FT size 131072
Total time 53 min, 7 sec
133.013
123.208
77.585
77.160
76.735
65.508
14.311
11.605
146.733
144.422
140.738
131.700
117.805
150
140
130
120
110
100
90
80
70
60
50
40
30
20
ppm

5d ^{1}H-NMR

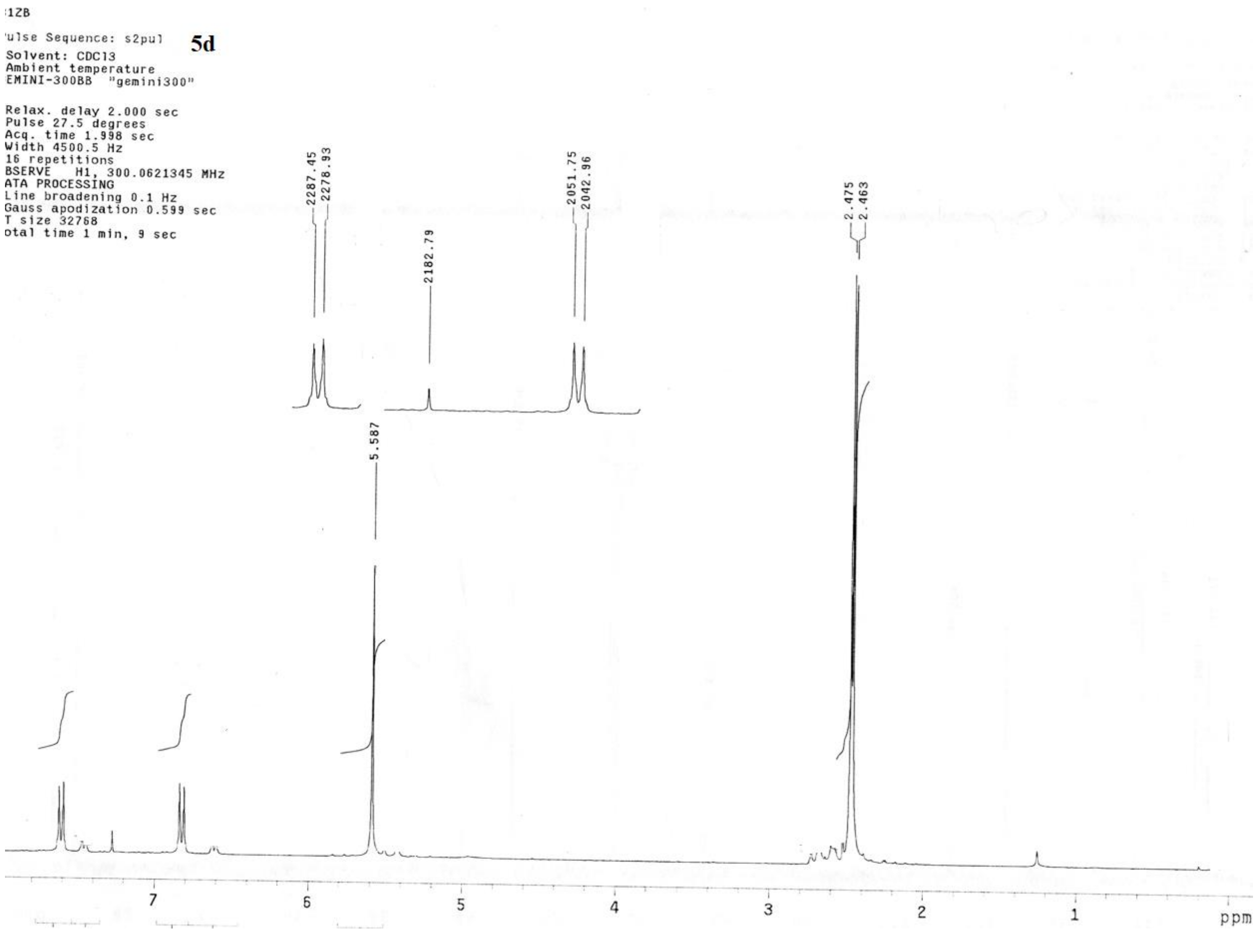

1ZB
ulse Sequence: s2pul
5d
Solvent: CDCl3
Ambient temperature
EMINI-300BB "gemini300"
Relax. delay 2.000 sec
Pulse 27.5 degrees
Acq. time 1.998 sec
Width 4500.5 Hz
16 repetitions
BSERVE H1, 300.0621345 MHz
ATA PROCESSING
Line broadening 0.1 Hz
Gauss apodization 0.599 sec
T size 32768
otal time 1 min, 9 sec
2287.45
2278.93
2182.79
2051.75
2042.96
2.475
2.463
5.587
7
6
5
4
3
2
1
ppm

5d ^{13}C-NMR

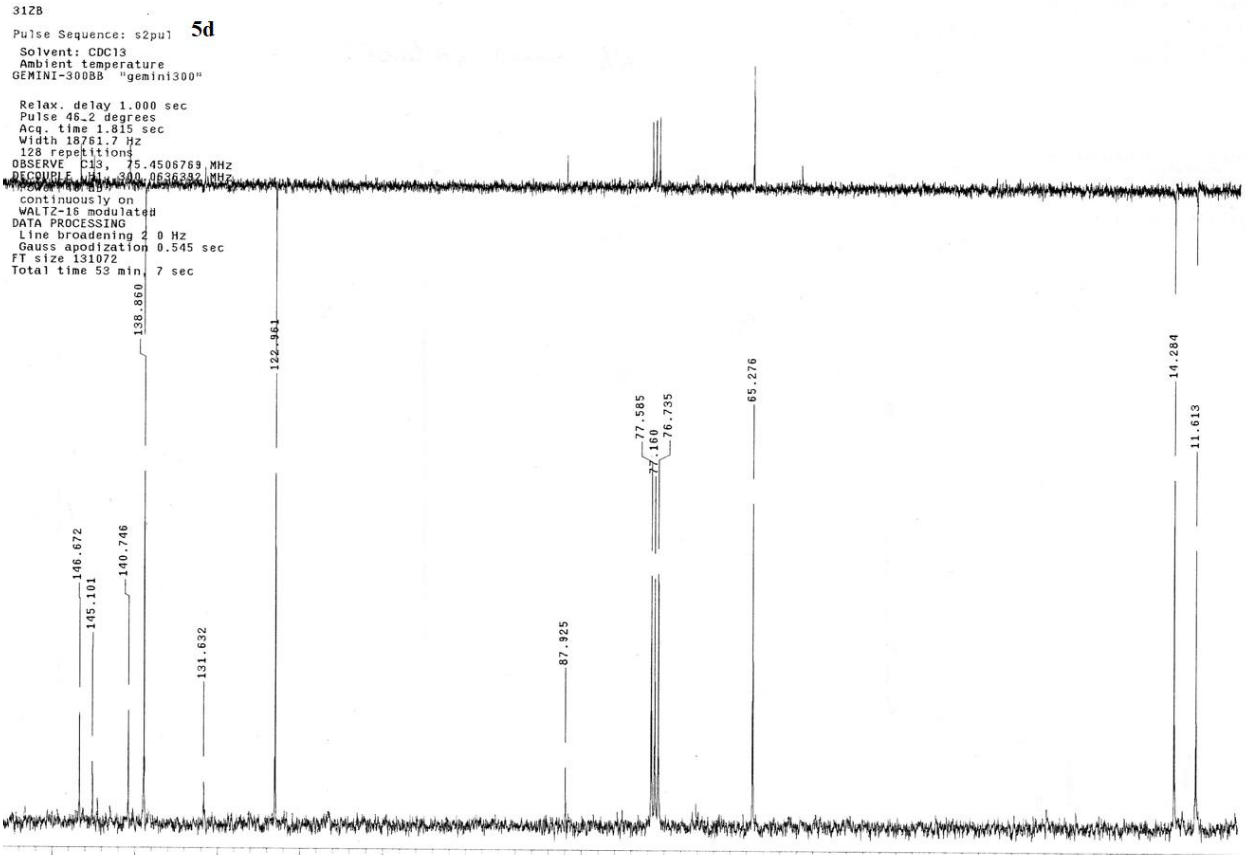
312B
Pulse Sequence: s2pul
5d
Solvent: CDCl3
Ambient temperature
GEMINI-300BB "gemini300"
Relax. delay 1.000 sec
Pulse 46.2 degrees
Acq. time 1.815 sec
Width 18761.7 Hz
128 repetitions
OBSERVE C13, 75.4506769 MHz
DECOUPLE H1, 300.0636392 MHz
continuously on
WALTZ-16 modulated
DATA PROCESSING
Line broadening 2.0 Hz
Gauss apodization 0.545 sec
FT size 131072
Total time 53 min, 7 sec
146.672
145.101
140.746
138.860
131.632
122.961
87.925
77.585
77.160
76.735
65.276
14.284
11.613

5e ¹H-NMR

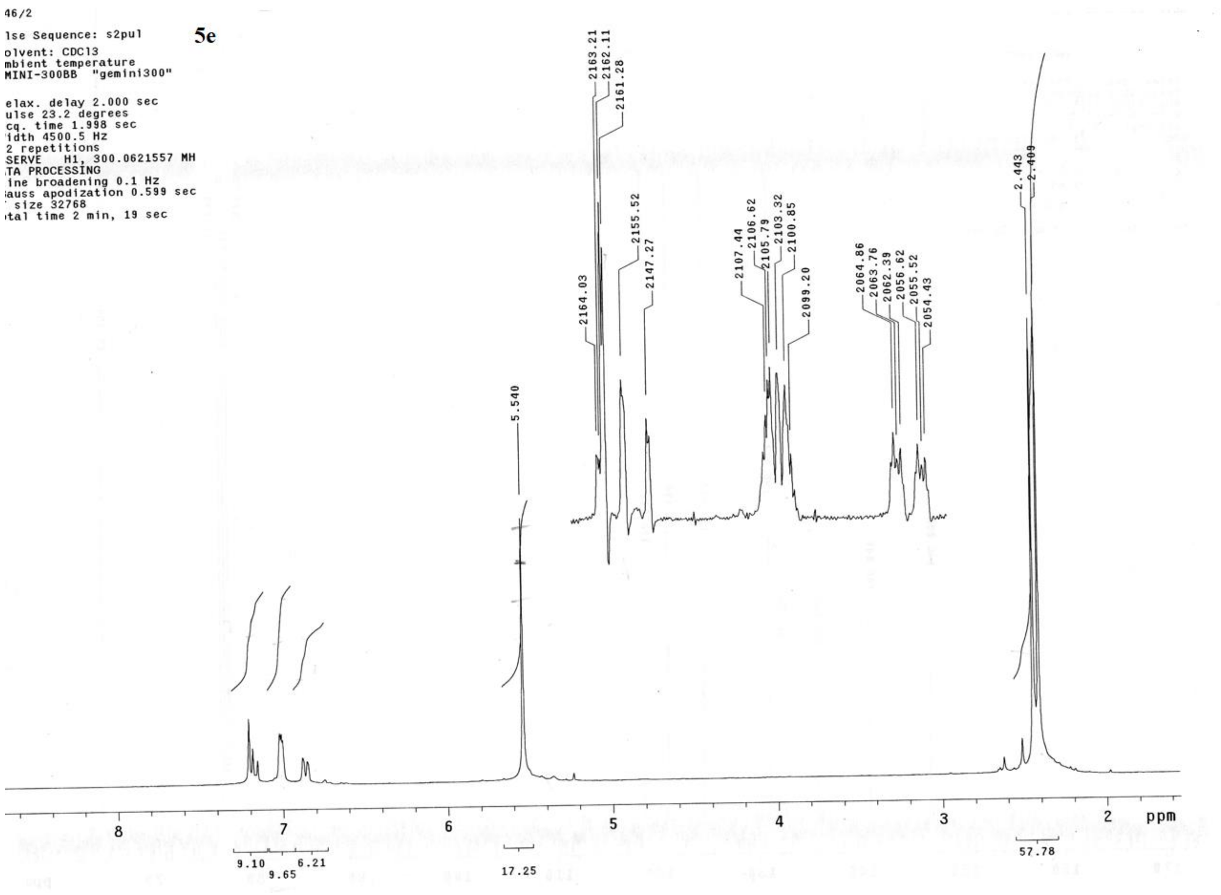
46/2
lse Sequence: s2pul
olvent: CDCl3
mbient temperature
MINI-300BB "gemini300"
elax. delay 2.000 sec
ulse 23.2 degrees
cq. time 1.998 sec
idth 4500.5 Hz
2 repetitions
SERVE H1, 300.0621557 MH
TA PROCESSING
ine broadening 0.1 Hz
auss apodization 0.599 sec
size 32768
tal time 2 min, 19 sec
5e
2163.21
2162.11
2161.28
2164.03
2155.52
2147.27
2107.44
2106.62
2105.79
2103.32
2100.85
2099.20
2064.86
2063.76
2062.39
2056.62
2055.52
2054.43
5.540
2.443
2.409
8
7
6
5
4
3
2
ppm
9.10
9.65
6.21
17.25
57.78

5e ^{13}C-NMR

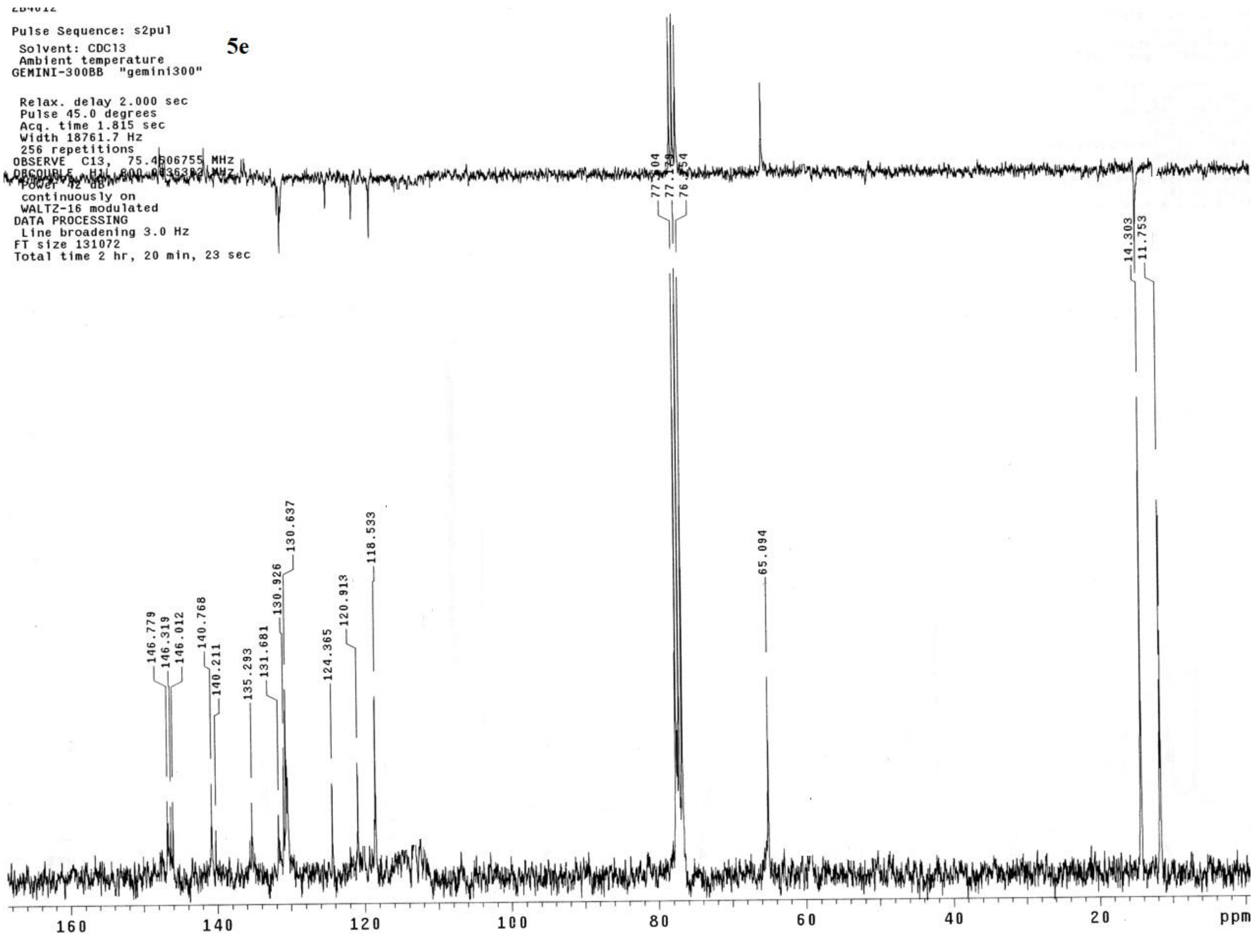
Pulse Sequence: s2pul
Solvent: CDCl3
Ambient temperature
GEMINI-300BB "gemini300"
5e
Relax. delay 2.000 sec
Pulse 45.0 degrees
Acq. time 1.815 sec
Width 18761.7 Hz
256 repetitions
continuously on
WALTZ-16 modulated
DATA PROCESSING
Line broadening 3.0 Hz
FT size 131072
Total time 2 hr, 20 min, 23 sec
14.303
11.753
146.779
146.319
146.012
140.768
140.211
135.293
131.681
130.926
130.637
124.365
120.913
118.533
65.094
160
140
120
100
80
60
40
20
ppm

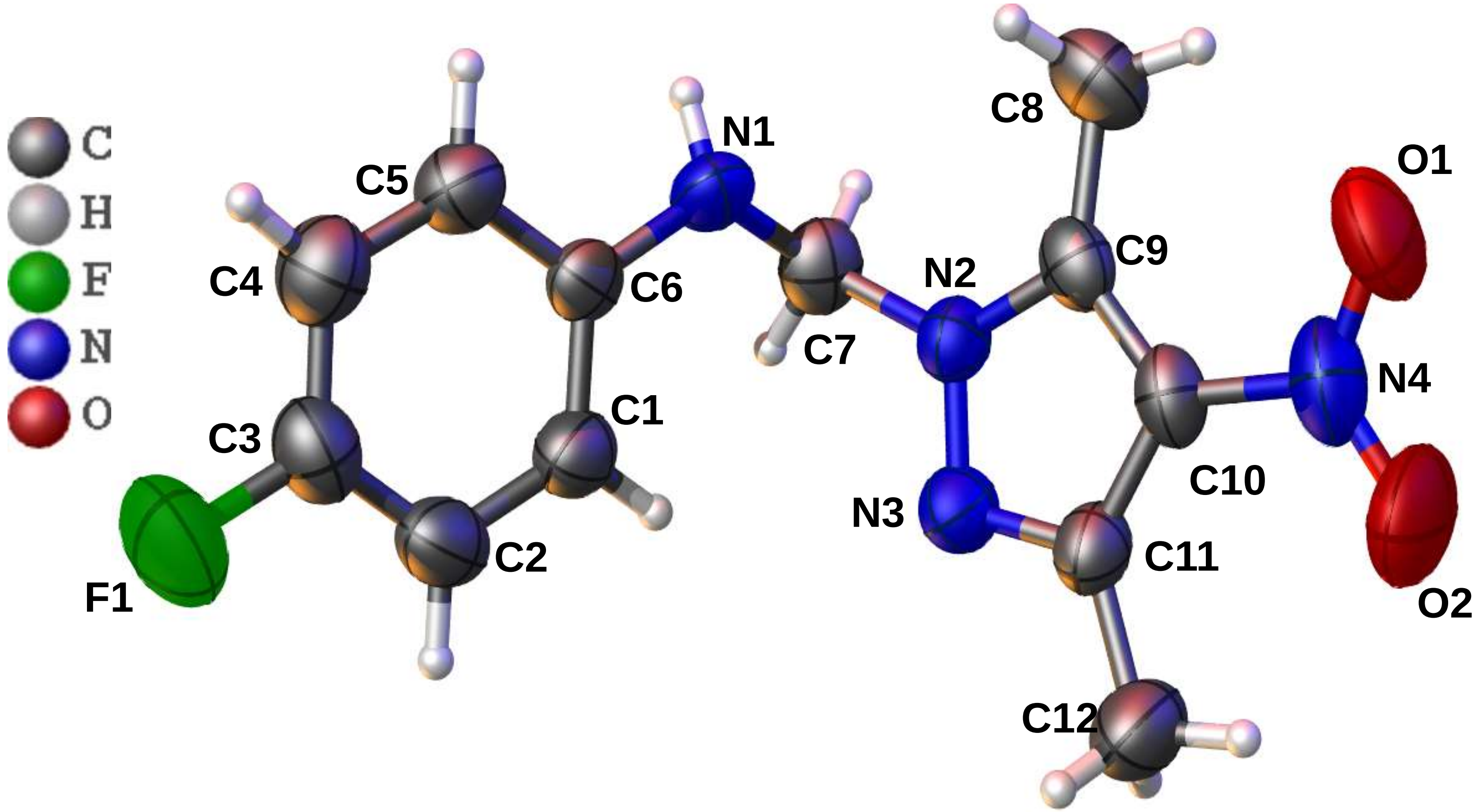

Figure S1. X-ray molecular structure of (**4a**) with color and atoms numbering scheme. Thermal ellipsoids representation with 50% probability.

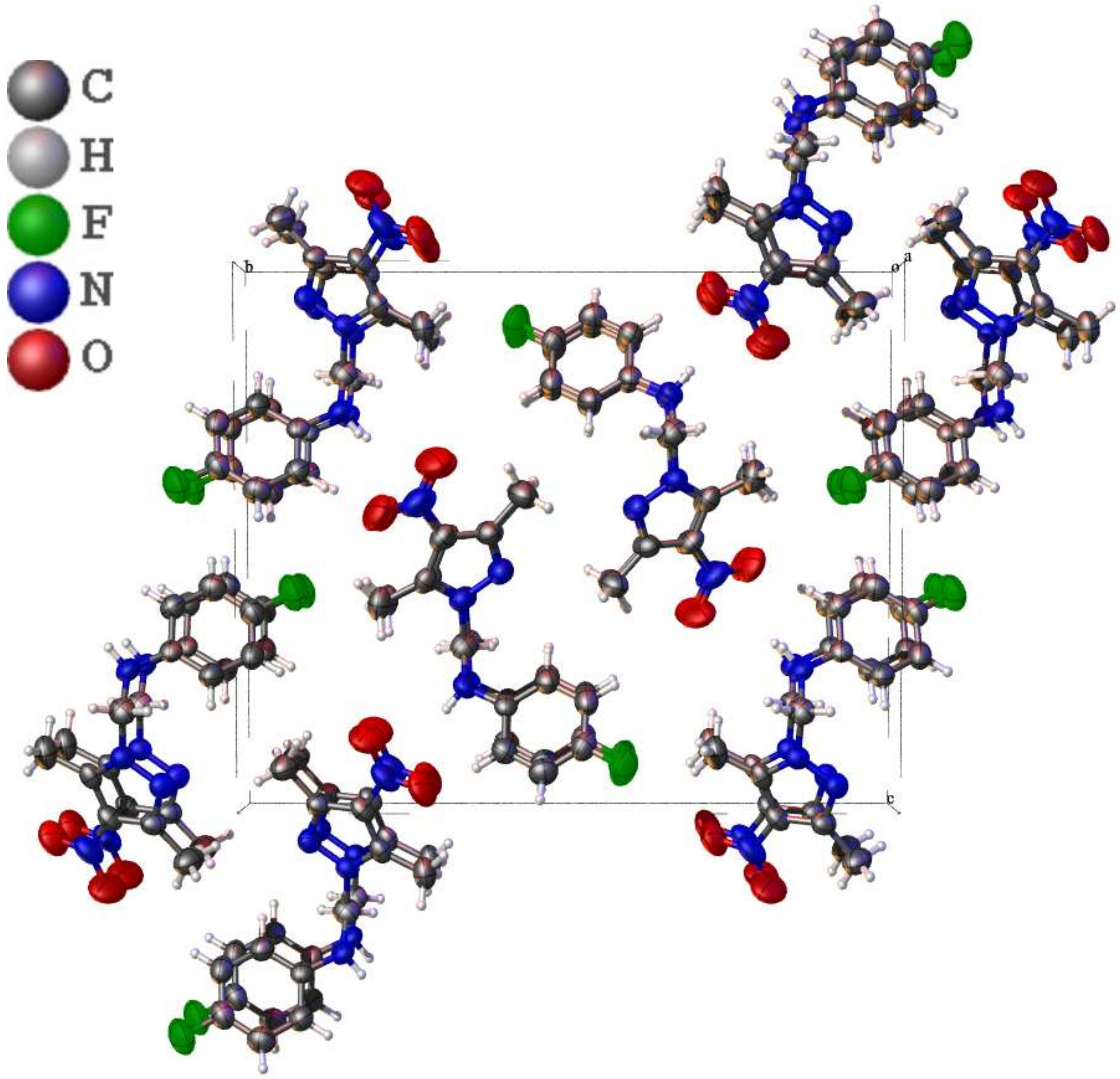

Figure S1.1. Crystal packing for (**4a**) along *a* axis.

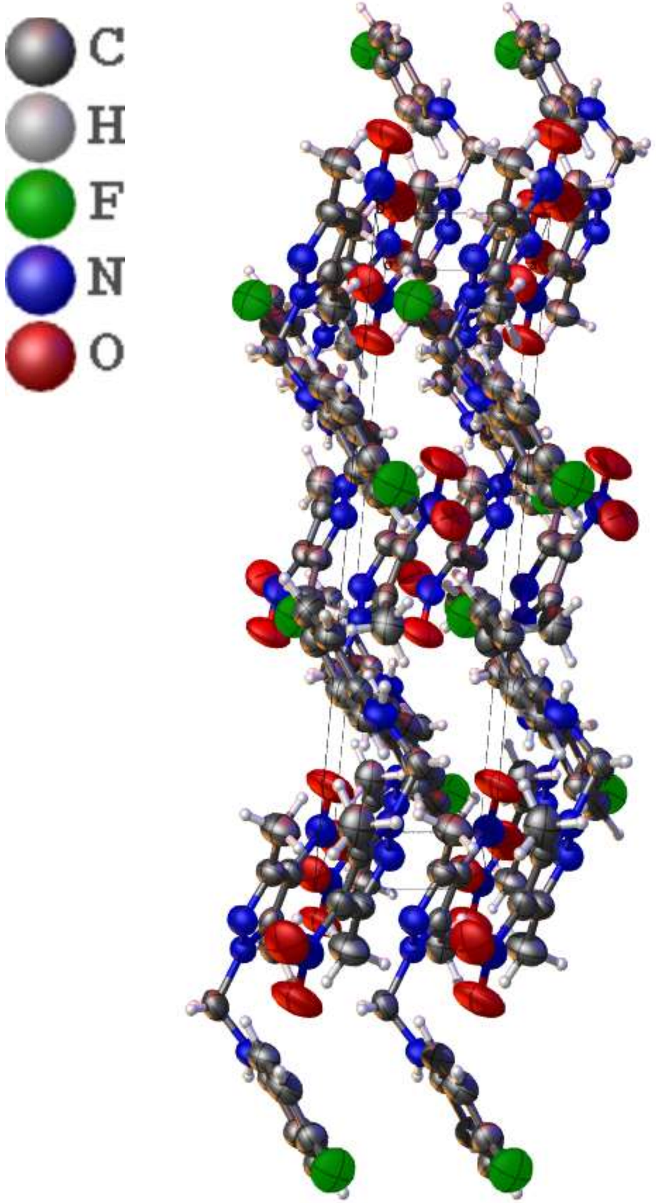

Figure S1.2. Crystal packing for (**4a**) along *b* axis.

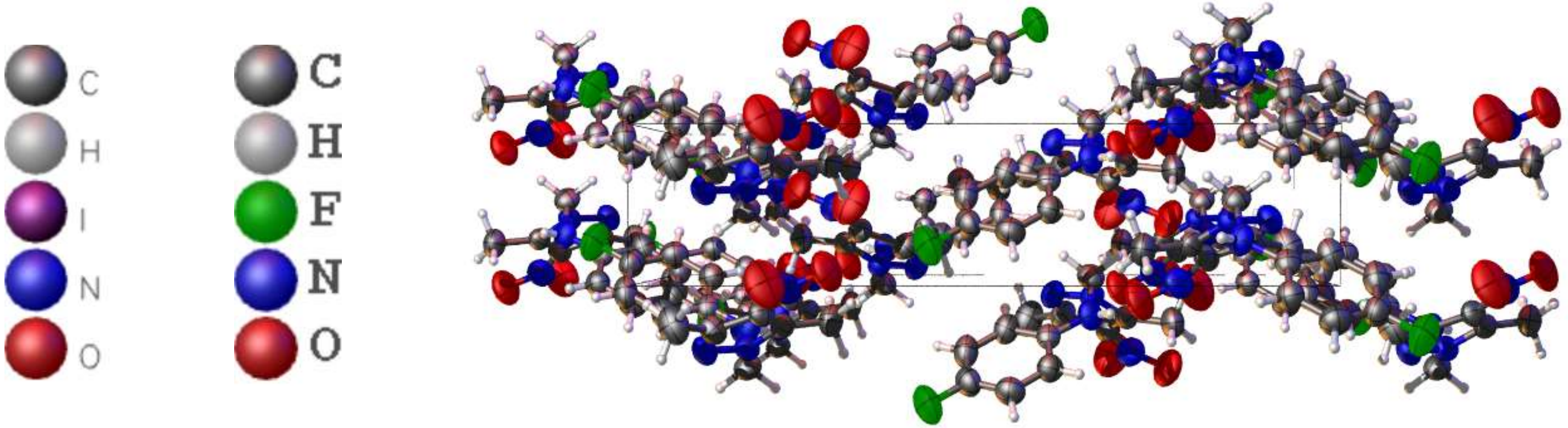

Figure S1.3. Crystal packing for (**4a**) along *c* axis.

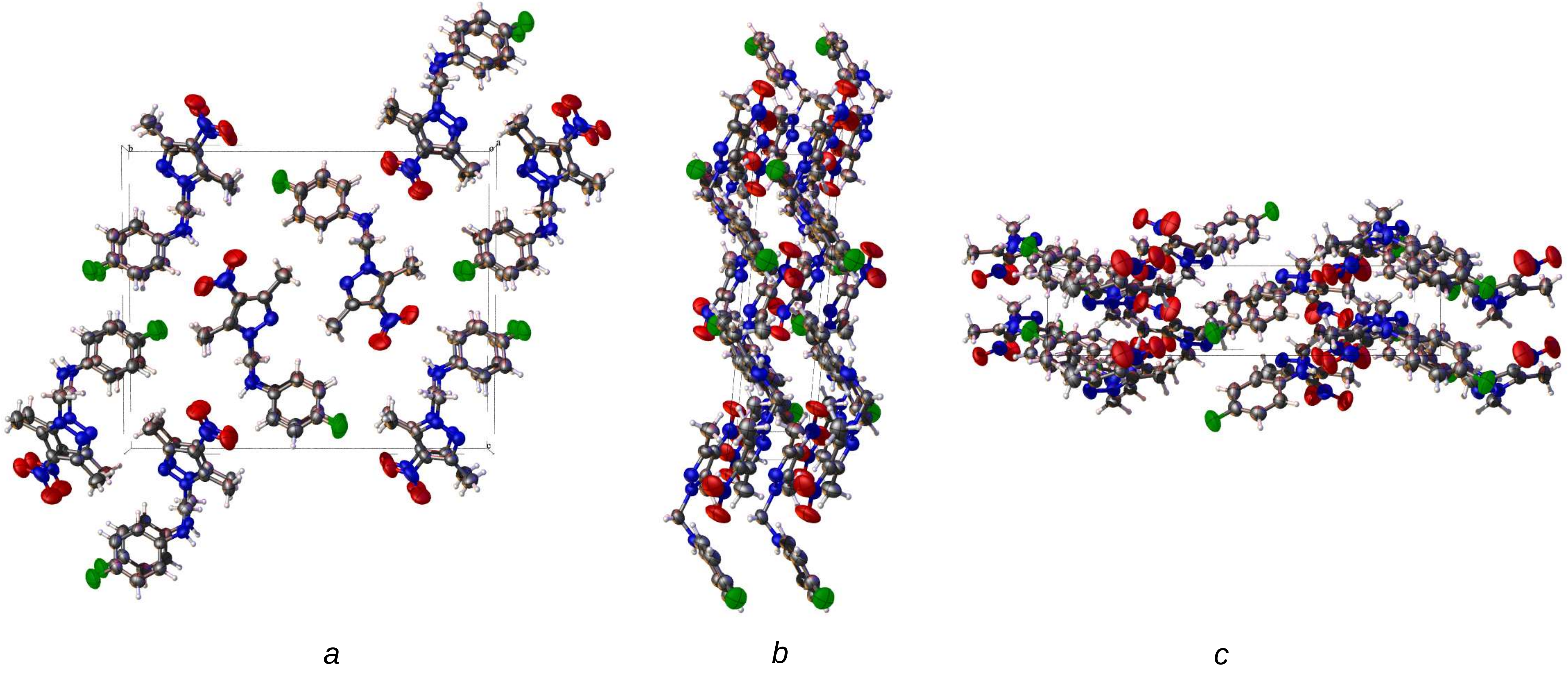

Figure S1.4. Crystal packing for (**4a**) along *a, b* and *c* axes.

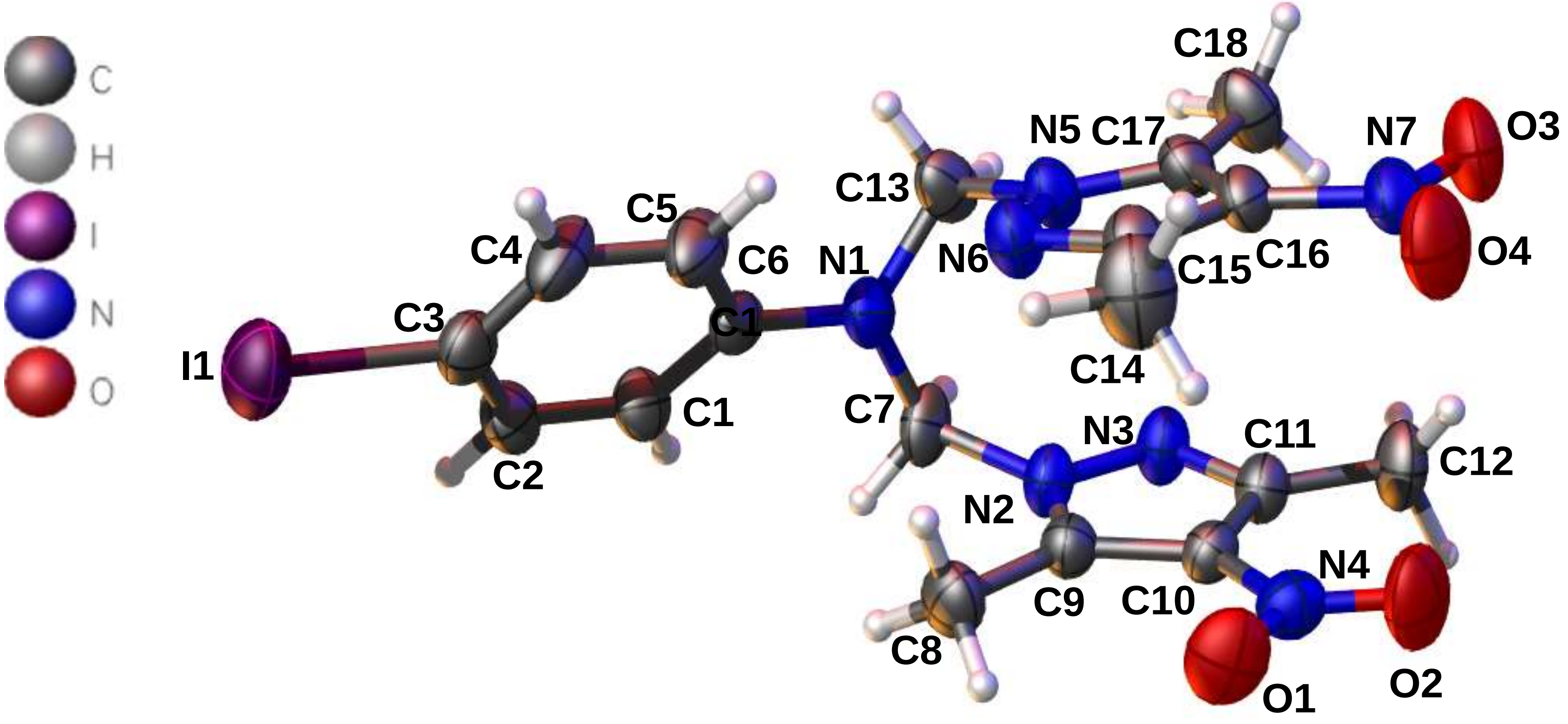

Figure S2. X-ray molecular structure of (**5d**) with color and atoms numbering scheme. Thermal ellipsoids representation with 50% probability.

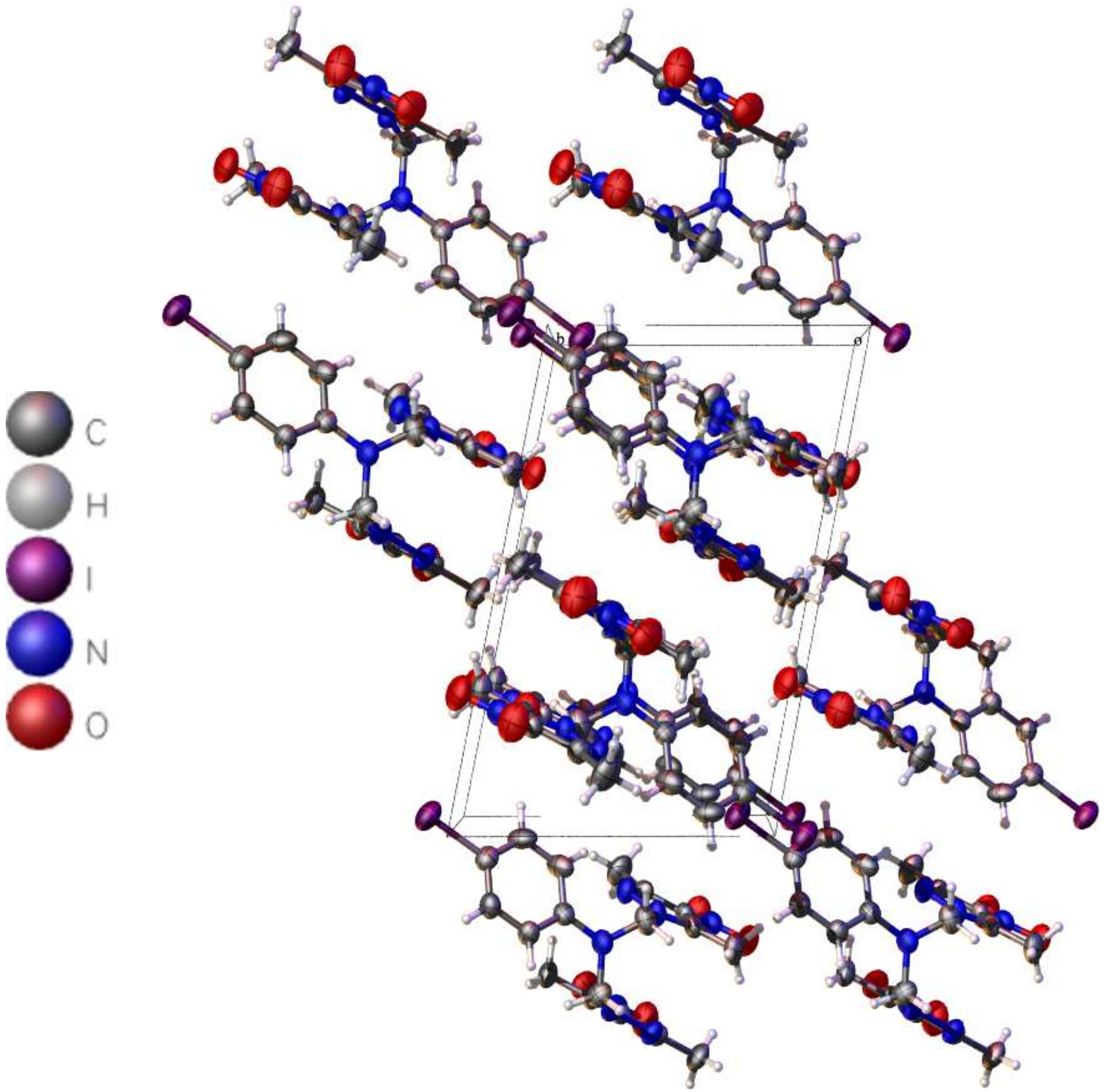

Figure S2.1. Crystal packing for (**5d**) along *a* axis.

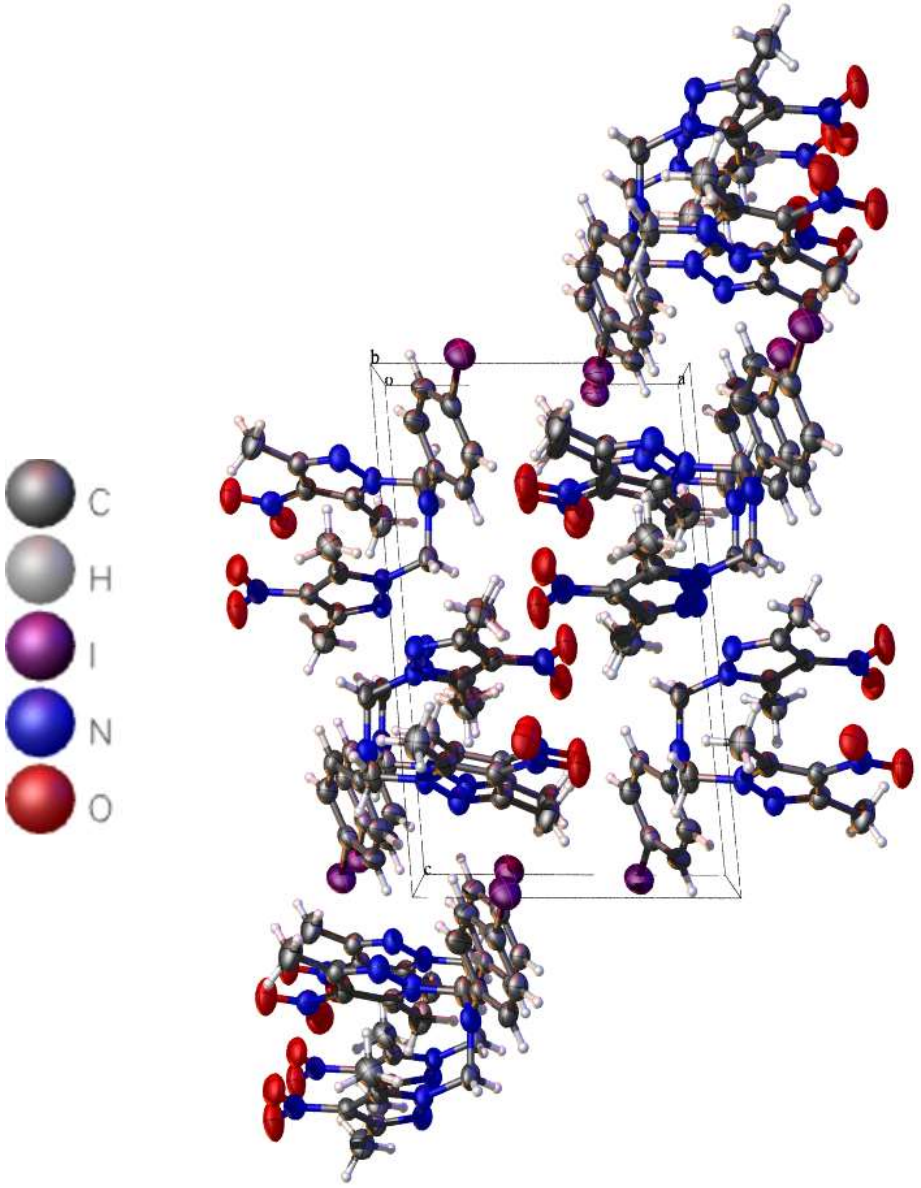

Figure S2.2. Crystal packing for (**5d**) along *b* axis.

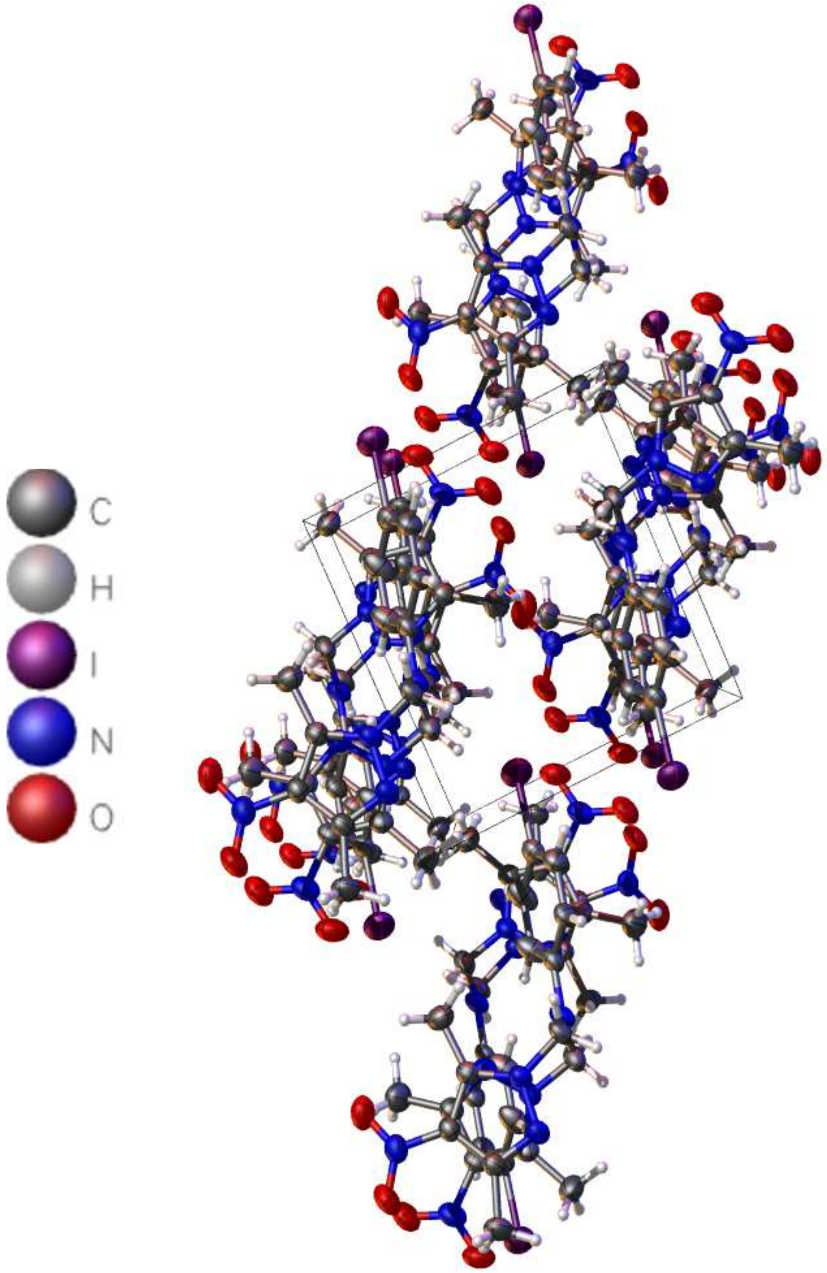

Figure S2.3. Crystal packing for (**5d**) along *c* axis.

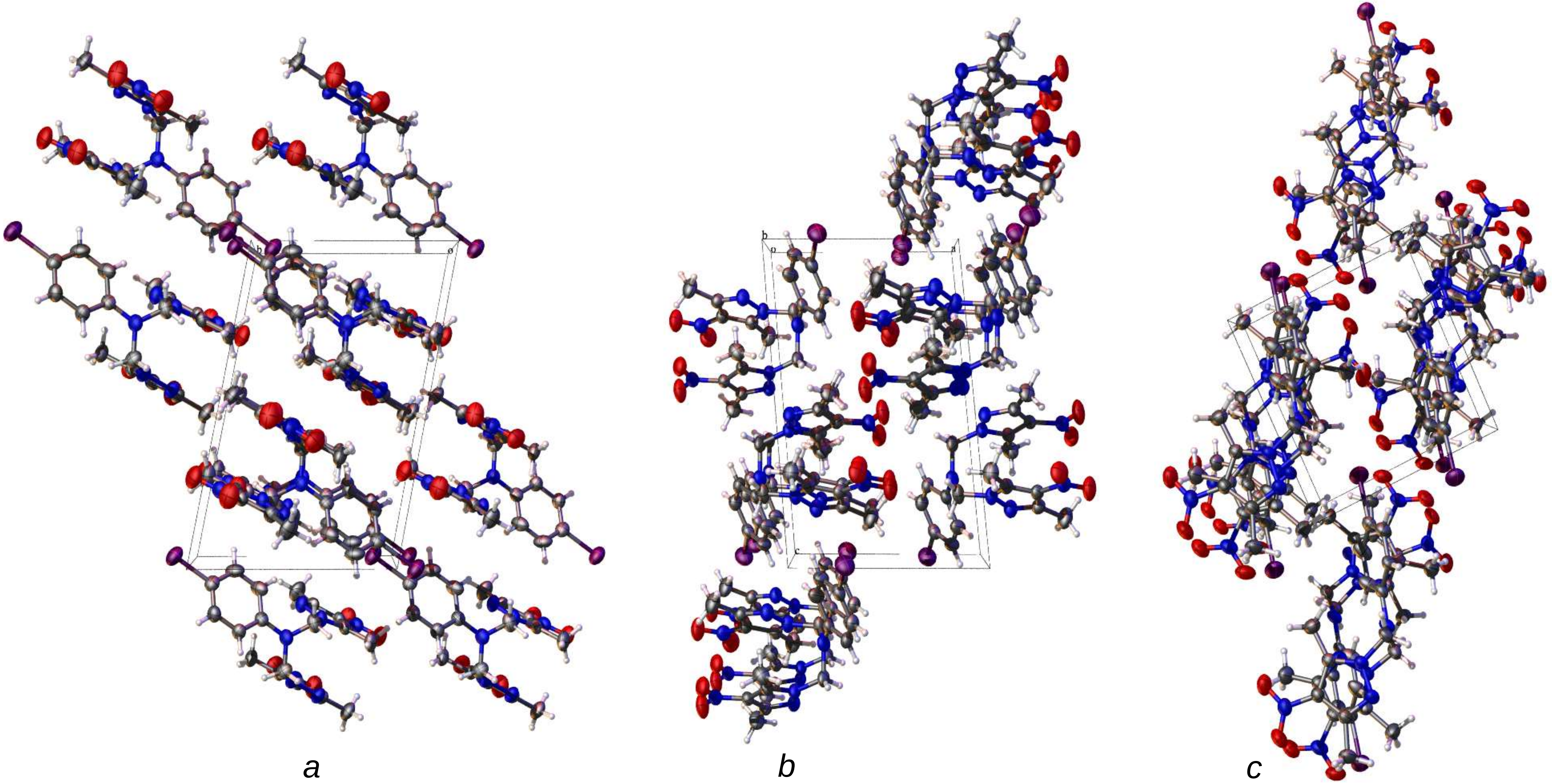

a *b* *c*

Figure S2.4. Crystal packing for (**5d**) along *a, b* and *c* axes.

Table S1. Summary of crystallographic data and refinement for **4a** and **5d.**

Crystal data	**4a**	**5d**
Empirical formula	$C_{12}H_{13}FN_4O_2$	$C_{18}H_{20}IN_7O_4$
Formula weight	264.261	525.309
Crystal size (mm)	0.300 x 0.100 x 0.100	0.450 x 0.400 x 0.300
Crystal system	Monoclinic	Triclinic
Space group	$P2_1/n$	P-1
a (Å)	4.2196(3)	8.5652(2)
b (Å)	19.7021(13)	9.0137(2)
c (Å)	15.3651(10)	13.5854(9)
α (°)	90	78.245(6)
β (°)	95.511(7)	84.279(6)
γ (°)	90	87.549(6)
Volume (Å^3)	1271.47(15)	1021.47(8)
Z	4	2
D calc.(g/cm^3)	1.380	1.708
F(000)	552.3	523.438
μMoK$_\alpha$ (cm^{-1})	0.107	1.609
Temperature (K)	293	293.15
Range of h, k, l	5, 23,19	9,11,17
θ min/max	3.40/27.40	3.02/27.49
Reflections collected/unique/observed	7698/2914/2914	14337/4682/4239
Data/restraints/parameters	2914/0/174	4682/30/275
Goodness of fit on F^2	0.981	1.0255
Final R indices [I>2σ(I)]	R_1=0.0576, wR_2=0.1243	R_1=0.0388, wR_2=0.1130
R indices (all data)	R_1=0.1452, wR_2=0.1672	R_1=0.0416, wR_2=0.1169

Table S2. Selected bond lengths (Å) and angles (°) for **4a** and **5d**.

**4a**		**5d**	
Atoms/Bond lengths (Å)	Atoms/Bond angles (°)	Atoms/Bond lengths (Å)	Atoms/Bond angles (°)
N2-N3 1.373(3	C11-N2-N3	I1-C3 2.096(3)	C9-N2-N3113.3(2)
N4-O2 1.226(3)	113.5(2)C10-	N7-C16 1.432(3)	C4-C5-C6121.1(3)
N2-C11 1.333(3)	C11-N2104.0(2)	N2-N3 1.372(3)	C7-N2-N3117.8(2)
N4-O1 1.228(3)	C7-N2-N3	C10-C9 1.388(4)	C10-C9-N2104.1(2)
N2-C7 1.476(3)	117.7(2)C12-	N2-C9 1.341(4)	C7-N2-C9128.0(2)
C10-C111.381(4)	C11-N2122.8(3)	C10-C11 1.412(4)	C8-C9-N2122.8(3)
F1-C3 1.372(3)	C7-N2-C11128.7(2)	N2-C7 1.470(3)	C10-N4-O1117.8(3)
C10-C9 1.402(4)	C12-C11-C10133.2(3)	C6-C5 1.383(5)	C8-C9-C10133.1(3)
N1-C6 1.402(3)	C7-N1-C6123.0(2)	N4-O1 1.225(4)	O2-N4-O1124.0(3)
C11-C121.485(4)	C1-C2-C3 118.6(3)	C6-C1 1.391(4)	N1-C7-N2111.9(2)
N1-C7 1.421(3)	C9-N3-N2105.4(2)	N4-C10 1.436(3)	O2-N4-C10118.2(3)
C2-C3 1.362(4)	C3-C4-C5 118.7(3)	N6-C15 1.321(4)	C2-C1-C6121.2(3)
N3-C9 1.325(3)	C5-C6-N1122.8(3)	N4-O2 1.198(4)	C11-N3-N2105.9(2)
C2-C1 1.365(4)	C10-C9-N3108.6(2)	C14-C15 1.491(4)	C18-C17-N5124.5(3)
C6-C5 1.379(4)	C1-C6-N1	N3-C11 1.318(3	N6-N5-C13117.9(2)
C4-C5 1.380(4)	118.6(2)C8-C9-	C5-C4 1.386(5)	C16-C17-N5104.3(2)
C6-C1 1.380(4)	N3120.3(3)	O4-N7 1.219(4)	C17-N5-C13129.1(3)
C4-C3 1.362(4)	C1-C6-C5	C9-C8 1.487(4)	C16-C17-C18131.2(3)
N4-C10 1.419(3)	118.6(3)C8-C9-	N5-C13 1.486(3)	C17-N5-N6113.1(2)
C9-C8 1.485(4)	C10131.0(2)	C1-C2 1.383(4)	C3-C4-C5119.8(3)

	O2-N4-C10 118.9(3)C4-C5-C6120.5(3)<br>O1-N4-C10 119.1(3)C2-C3-F1 118.9(3)<br>O1-N4-O2122.0(3)<br>C4-C3-F1 118.9(3)<br>C11-C10-N4125.7(3)<br>C4-C3-C2122.2(3)<br>C9-C10-N4125.8(3)<br>C2-C1-C6121.4(3)<br>C9-C10-C11108.5(2)<br>N1-C7-N2 113.0(2)	N5-N6 1.377(3)<br>C17-C181.486(4)<br>N5-C17 1.337(4)<br>C17-C161.388(4)<br>N1-C13 1.428(4)<br>C4-C3 1.385(5)<br>N1-C6 1.415(4)<br>C11-C12 1.489(4)<br>N1-C7 1.445(4)<br>C15-C161.404(4)<br>N7-O3 1.216(4)<br>C2-C3 1.376(5)	C6-N1-C13120.9(3)<br>C10-C11-N3108.9(2)<br>C7-N1-C13117.7(3)<br>C12-C11-N3120.6(3)<br>C7-N1-C6121.4(2)<br>C12-C11-C10130.4(3)<br>O3-N7-O4123.1(3)<br>C14-C15-N6120.6(3)

Table S3. Cell viability assay results of series **4a-f** tested *in vitro* on NCTC normal fibroblasts and HEp-2 epitelial tumor cells, at 24 and 48h of treatment

**Sample**	**Concentration (μg/mL)**	**Cell viability on NCTC cells (%)**		**Cell viability on Hep2 cells (%)**	
		24h	48h	24h	48h
**Control**		100.00	100.00	100.00	100.00
**4a**	3.1	99.56±1.38	110.87±3.28	99.14±3.42	93.05±0.78
	6.25	99.35±2.20	109.13±3.80	94.44±1.19	89.00±1.50
	12.5	99.02±2.51	100.63±1.48	93.38±0.92	83.79±2.30
	25	90.22±1.71	73.46±2.96	91.35±3.73	72.26±1.99
	50	62.72±1.88	47.48±1.31	73.18±2.71	36.37±1.45
**4b**	3.1	100.17±1.63	95.08±0.82	115.84±2.48	112.57±1.03
	6.25	100.53±3.31	93.03±1.35	105.08±2.91	104.00±1.08
	12.5	98.60±2.62	89.70±3.15	100.30±2.49	98.02±1.42
	25	96.31±2.22	61.85±1.38	88.79±1.65	79.53±1.49
	50	64.91±0.94	31.57±1.27	65.62±2.78	46.82±3.35
**4c**	3.1	99.20±0.97	99.67±0.71	101.36±2.04	116.32±2.55
	6.25	97.10±3.04	98.85±1.92	102.66±2.31	104.22±0.88
	12.5	95.60±0.62	97.21±3.50	100.48±3.15	99.57±2.32
	25	95.50±2.14	95.64±2.93	98.70±1.83	82.62±3.28
	50	91.11±3.04	81.53±3.26	92.64±0.90	70.23±1.91
**4d**	3.1	99.89±2.31	95.69±0.80	104.97±1.93	100.60±3.72
	6.25	100.53±2.24	96.26±0.78	109.78±2.64	110.14±3.49
	12.5	102.74±3.44	92.64±1.40	113.02±3.48	110.79±2.80
	25	101.67±1.07	91.55±1.41	109.40±2.75	101.56±0.56
	50	101.37±2.55	88.16±2.10	106.24±1.63	68.97±0.81
**4e**	3.1	93.11±2.76	89.55±2.24	106.54±0.73	98.12±2.88
	6.25	94.28±2.92	98.33±2.03	103.71±0.28	97.11±1.78
	12.5	97.81±0.99	93.18±2.94	99.70±0.32	94.75±2.84
	25	95.63±2.19	92.20±0.89	89.15±0.88	81.69±2.38
	50	63.53±3.18	63.21±2.56	56.46±0.42	54.91±2.76
**4f**	3.1	107.06±1.61	99.61±1.20	98.29±1.16	98.26±3.38
	6.25	107.50±1.60	105.98±1.70	98.61±0.72	97.74±0.62
	12.5	103.04±1.05	103.07±0.68	95.51±2.45	93.63±0.75
	25	93.69±2.77	97.79±2.35	94.34±2.78	86.05±0.93
	50	87.72±1.77	80.47±1.16	93.91±1.03	72.84±2.33

Table S4. Cell viability assay results of series **5a-e** tested *in vitro* on NCTC normal fibroblasts and HEp-2 epitelial tumor cells, at 24 and 48h of treatment

**Sample**	Concentration (µg/mL)	Cell viability on NCTC cells (%)		Cell viability on Hep2 cells (%)	
		24h	48h	24h	48h
**Control**		100.00	100.00	100.00	100.00
**5a**	3.1	97.69±1.80	87.19±0.70	90.85±1.10	90.09±2.22
	6.25	98.35±2.22	88.74±2.40	104.37±0.32	85.25±3.13
	12.5	100.00±2.07	98.18±2.04	96.62±3.10	80.09±1.54
	25	97.36±2.40	93.71±1.17	82.68±1.85	67.37±3.23
	50	88.56±2.66	92.55±1.32	57.18±1.13	38.80±0.71
**5b**	3.1	99.08±3.42	100.38±2.53	105.41±1.52	110.12±1.25
	6.25	103.36±3.06	94.93±2.14	103.69±2.25	94.67±3.80
	12.5	98.78±3.47	84.05±3.77	101.68±3.76	87.08±0.92
	25	70.03±2.51	55.16±1.60	90.80±2.84	74.80±0.33
	50	48.62±1.35	28.14±0.35	65.27±2.02	54.56±1.17
**5c**	3.1	104.64±1.96	96.74±2.96	111.76±2.32	96.94±1.38
	6.25	106.01±2.30	101.14±3.63	113.54±2.18	96.48±1.58
	12.5	105.90±3.74	97.37±1.99	116.63±2.83	89.21±2.02
	25	105.37±1.88	96.63±2.02	112.94±1.52	65.31±2.09
	50	83.98±2.12	68.00±1.61	101.73±2.71	50.25±2.02
**5d**	3.1	102.67±1.49	93.58±0.71	88.45±0.28	96.17±0.13
	6.25	107.86±2.40	103.10±3.22	89.15±0.73	78.61±0.67
	12.5	106.29±2.05	101.77±3.03	91.41±0.69	61.51±0.34
	25	98.43±3.87	95.80±2.12	89.86±0.48	45.75±0.33
	50	83.02±2.90	88.27±1.55	89.86±0.16	20.84±0.13
**5e**	3.1	101.94±0.73	95.71±0.50	103.69±0.73	102.47±0.48
	6.25	98.57±0.27	89.47±0.34	102.40±0.28	96.06±0.34
	12.5	88.26±0.32	69.25±0.30	82.29±0.32	77.40±0.63
	25	77.65±0.88	40.30±0.68	70.51±0.88	60.32±0.67
	50	65.79±0.42	26.45±0.46	59.94±0.42	37.74±0.46
**dioscin**	3.1	94.29±2.49	94.17±1.66	97.09±0.43	95.99±0.53
	6.25	93.04±3.24	72.63±1.67	76.68±2.01	61.97±1.78
	12.5	38.74±2.55	37.02±2.55	58.92±0.30	38.03 ±2.09
	25	14.93±2.63	8.55±1.26±	13.86±0.18	7.49±1.32
	50	13.73±2.06	7.14±1.28	11.53±0.14	7.27±1.14

Legend: the green color represents the non-cytotoxic effect, cell viability percentage>80%. the blue color represents the slightly cytotoxic effect, percentage of cell viability between 50 and 80%. the red color represents the severe cytotoxic effect, percentage of cell viability < 50 %.
